# Supplementary material for: Exploration of the proxiOME of large subunit ribosomal proteins reveals Acl1 and Bcl1 as cooperating dedicated chaperones of Rpl1
Source: Nucleic Acids Res. 2026 Mar 30;54(6):gkag264. doi: 10.1093/nar/gkag264 (PMC13034042; doi:10.1093/nar/gkag264)
Supplement: gkag264_Supplemental_Files [file gkag264_supplemental_files.zip › SupplementaryData.pdf]

Supplementary Data

Exploration of the proxiOME of large subunit ribosomal proteins reveals Acl1 and Bcl1 as cooperating dedicated chaperones of Rpl1

Sébastien Favre[1], Benjamin Pillet[1], Fabiana Burchert[2,†], Devanarayanan Siva Sankar[1,†], Alfonso Méndez-Godoy[1], Stephan Kiontke[2], Jörn Dengjel[1], Gert Bange[2,3], and Dieter Kressler[1,*]

[1] Department of Biology, University of Fribourg, 1700 Fribourg, Switzerland

[2] Center for Synthetic Microbiology (SYNMIKRO) and Department of Chemistry, Philipps-University Marburg, 35043 Marburg, Germany

[3] Max Planck Institute for Terrestrial Microbiology, Molecular Physiology of Microbes, 35043 Marburg, Germany

[*]To whom correspondence should be addressed. Email: dieter.kressler@unifr.ch

[†]Fabiana Burchert and Devanarayanan Siva Sankar contributed equally to this work.

Present address: Devanarayanan Siva Sankar, Global Health Institute, Swiss Federal Institute of Technology Lausanne (EPFL), 1015 Lausanne, Switzerland

The Supplementary Data includes:

Supplementary Notes 1 and 2

Supplementary Figures S1 to S19

Supplementary Table S1

Supplementary References

Supplementary Note 1: Neighbourhoods of r-proteins on pre-ribosomal particles

While the specific enrichment of the AFs Mak11, Nog1, Rlp24, Rrp15, Spb4, and Ssf1/2 can be readily explained by their immediate proximity to Rpl3's C-terminal end in the available pre-60S cryo-EM structures, the TurboID assays with the Rpl3-TurboID bait also indicated potential proximities with the reproducibly and highly enriched AFs Dbp9 and Loc1 (Fig. 1B and C and Supplementary Fig. S3). The DEAD-box RNA helicase Dbp9 is a substoichiometric component of primordial and early nucleolar pre-60S particles but is not visible in the cryo-EM structures of early nucleolar, Nsa1-defined pre-60S particles (state 2 and states A, B, and C) [1-4]; however, as Dbp9 and Rpl3 are functionally connected by a synthetic lethal relationship and as crosslinking MS revealed a potentially direct interaction between Dbp9 and the Rpl3-proximal r-protein Rpl9 (uL6) [5,6], the observed enrichment of Dbp9 in the Rpl3-TurboID assay likely reflects a true physical proximity. In further support of this possibility, the proximity labelling with N-terminally TurboID-tagged Rlp24 also revealed a substantial enrichment of Dbp9 (Supplementary Figs. S1 and S3); notably, the C-terminal end of Rpl3 and the N-terminal end of the adjacent Rlp24 project in a similar direction from the pre-60S surface [2]. In the case of Loc1, the available cryo-EM structures of pre-60S particles do not offer a straightforward explanation for its suggested proximity to Rpl3's C-terminal end. While Loc1 is already substantially enriched in early and/or intermediate nucleolar pre-60S particles sequentially purified via the Nsa1 and Ytm1 baits [4], and a short stretch of Loc1 could recently be visualized on early nucleolar, Ssf1-defined pre-60S particles [7], a reasonable portion of Loc1 only starts to be visible in late nucleolar state E pre-60S particles purified via the Spb4 bait [8,9]. On these state E pre-60S particles, the modelled Loc1 segments (residues 10-63 and 74-148) are located on the opposite side of Rpl3 (Supplementary Fig. S4A); however, it cannot be excluded that parts of Loc1, especially its lysine-rich C-terminal region (residues 149-204), which is not resolved in the state E pre-60S particles and projects away from the pre-60S surface, could be within the radius of the bioAMP cloud generated by the TurboID moiety emanating from Rpl3's C-terminus on earlier pre-60S particles.

Intriguingly, Rpl2 (uL2) is, after the Rpl3 bait protein, the most enriched r-protein in the Rpl3-TurboID assays (Fig. 1B and C). Rpl2 can for the first time be visualized on the early nucleoplasmic state NE1 pre-60S intermediate (Supplementary Fig. S4B; [8]); at this maturation stage, on subsequent pre-60S particles, and on the mature LSU, Rpl2 appears, however, to be too far away from Rpl3's C-terminus to explain its substantial enrichment. The visible incorporation of Rpl2, i.e., of its central, well-structured part (residues 25-200), and the neighbouring Rpl43 (eL43) is enabled by and/or coincides with the release of several AFs, including Loc1, Rrp17, Noc2, Noc3, and Spb4, which is part of the major compositional and structural rearrangements occurring during the transition from the late nucleolar state E to the early nucleoplasmic state NE1/NE2 pre-60S particles [8,10]. On the state E2 pre-60S intermediate, where the rRNA-binding sites of the central Rpl2 part within folding domains II and III are occluded by Noc2 and Noc3, respectively, the unresolved rRNA region encompassing helices H65 to H71, which contains the main rRNA-binding sites (H66 and H68) of Rpl2 and

participates in intersubunit bridges on mature 80S ribosomes [11], emanates next to the N-terminal part of Rrp17's long, surface-exposed α-helix from the surface (Supplementary Fig. S4A). We speculate that Rpl2 could already be associated, likely via its extensive interactions with rRNA helices H66 and H68, with the state E2 and/or during the transition to the state NE1 pre-60S intermediate. In this scenario, by being associated with this unresolved and, thus, presumably flexible rRNA region, Rpl2 might get into close enough proximity of Rpl3's C-terminus to account for its observed enrichment in the TurboID assays.

In support of the suitability of the TurboID-based proximity labelling approach for the detection of r-protein neighbourhoods on pre-60S particles, the single data set TurboID assays revealed transient proximities of LSU r-proteins with directly adjacent AFs that can also be observed in pre-60S cryo-EM structures. Nsa1, for example, was most enriched in the proximity labelling assays of the C-terminally TurboID-tagged Rpl26 (uL24) and the N-terminally TurboID-tagged Rpl35 (uL29), respectively (Supplementary Fig. S5A, left panel); these two r-proteins are in close vicinity of Nsa1 within early nucleolar pre-60S particles (state 2 and states A to D) [2,3], in both cases positioning the fused TurboID moiety on the pre-60S surface in reasonable proximity of Nsa1 (Supplementary Fig. S5A, right panel). Rrp1, another member of the four-component Nsa1 module, was found to be most strongly enriched in the assays with the N-terminally TurboID-tagged Rpl4, Rpl7 (uL30), and Rpl18 (eL18) baits (Supplementary Fig. S5B, upper panel). These three r-proteins are in immediate proximity and even in direct contact with Rrp1 from the earliest structurally characterized pre-60S assembly intermediate (the so-called Noc1-Noc2 RNP), on which Rrp1 can, contrary to Nsa1, already be visualized, until the state D pre-60S intermediate, from which the Nsa1 module gets, presumably concertedly, released by the AAA-ATPase Rix7 [1-3,12]. While the N-terminal 14 residues of Rpl18 are not resolved in the cryo-EM structures of these early nucleolar pre-60S intermediates, the surface-exposed N-termini of Rpl4 and Rpl7 are directly adjacent to Rrp1 (Supplementary Fig. S5B, lower panel). Nmd3 and Lsg1 were prominently enriched in the TurboID assays performed with C-terminally TurboID-tagged Ubi1 (Rpl40a) and N-terminally TurboID-tagged Rpl10 (Supplementary Fig. S5C, left panels), two r-proteins that only get assembled during the cytoplasmic pre-60S maturation phase and can first be visualized on the state III and state IV pre-60S intermediate, respectively [13]; thus, recapitulating their observed proximities on late cytoplasmic pre-60S intermediates purified via the Lsg1-TAP bait (Supplementary Fig. S5C, right panel). Intriguingly, Yvh1, which could only be visualized on the state I and state II pre-60S intermediates and, thus, prior to the visible incorporation of Rpl40 and Rpl10 [13], was also substantially enriched in these Rpl40 and Rpl10 TurboID assays (Supplementary Fig. S5D, upper panel), suggesting that Yvh1 is nevertheless simultaneously present with Rpl40 and/or Rpl10 on cytoplasmic pre-60S intermediates. Accordingly, the order of cytoplasmic maturation events might be less hierarchical than deduced from the cryo-EM snapshots of consecutive Lsg1-purified pre-60S intermediates [13]. In agreement with this possibility, cryo-EM structures of late cytoplasmic pre-60S intermediates purified via the Nmd3-TAP bait from cells treated with the Drg1 inhibitor diazaborine

revealed the simultaneous presence of Yvh1 and Rpl40 on pre-Lsg1 and Lsg1-engaged pre-60S particles and of Yvh1, Rpl40, and Rpl10 on the Rpl10-inserted pre-60S particle [14] (Supplementary Fig. S5D, lower panel). However, it cannot be excluded that partial inhibition of Drg1 or the fused TurboID moiety, especially in the case of Rpl40, could delay the release of Yvh1.

Supplementary Note 2: The proxiOME of Bcp1

The Bcp1 proxiOME also included an already validated binding partner of Rpl23 (Rkm1) and two potential direct interactors of Bcp1 (Kap60 and Mss4). The SET domain-containing methyltransferase Rkm1, which is responsible for dimethylation of lysine residues K106 and K110 of Rpl23 but dispensable for optimal growth of yeast cells [15-17], was shown to form a binary complex with Rpl23, but not with Bcp1, and a trimeric complex with Rpl23 and Bcp1 in vitro [17]. Moreover, co-immunoprecipitation experiments have indicated an Rpl23-dependent interaction between Bcp1 and Rkm1 [17]; thus, our proximity labelling data, showing an enrichment of Rkm1 in both the Rpl23 and Bcp1 TurboIDs (Supplementary Figs. S6F and S8E), provide further evidence for the in vivo occurrence of the trimeric Bcp1-Rpl23-Rkm1 complex. Bcp1 contains two predicted classical NLS regions [18], the first (residues 11-20) within its N-terminal extension and the second (residues 219-234) within an internal loop. According to AlphaFold3 predictions [19], positively charged side chains of the first and second NLS region interact in a canonical manner with the minor and major NLS-binding site [20], respectively, of the importin-α Kap60 (Supplementary Fig. S9A, left panels), suggesting that the transport adaptor Kap60, in conjunction with the importin-β Kap95, could mediate the nuclear import of Bcp1. Mss4 is an essential phosphatidylinositol-4-phosphate 5-kinase that almost exclusively localizes to the plasma membrane [21,22]. Interestingly, Mss4 also contains a functional NLS (residues 347-364), and overexpressed C-terminally GFP-tagged Mss4 exhibits, besides its normal location at the plasma membrane, a nuclear localization, which is no longer observed in cells lacking the importin-β Kap123 [22]. Moreover, the C-terminally GFP-tagged Mss4-1 mutant protein, bearing the D127N and L393P substitutions, was shown to almost exclusively accumulate in the nucleus at the non-permissive temperature. Notably, while absence of Kap123 abrogated the nuclear accumulation and conferred a cytoplasmic localization, overexpression of Bcp1 fully restored the plasma membrane localization of Mss4-1-GFP [22]. This data was, by also considering the finding that a temperature-sensitive *bcp1* allele (F241S) conferred a pre-60S export defect, interpreted as Bcp1 being responsible for nuclear export of Mss4 [22]. In line with their physical proximity suggested by the Bcp1 TurboID assays, AlphaFold3 predicted with good confidence the formation of a Bcp1-Mss4 complex (Supplementary Fig. S9A, right panels). According to this model, Bcp1 interacts with the first subdomain (residues 376-555) of the phosphatidylinositol phosphate kinase (PIPK) domain (residues 376-756) as well as with a segment (residues 335-357) that precedes the first PIPK subdomain and notably encompasses part of the NLS region of Mss4. Consistent with this predicted mode of interaction, an Mss4 variant (residues 332-565) consisting essentially of the N-terminally extended first PIPK subdomain showed a robust Y2H interaction with full-length Bcp1 (Supplementary Fig. S9B). Moreover, as an Mss4 variant (residues 371-565) comprising only the first PIPK subdomain exhibited a reduced Y2H interaction, it appears that both predicted Bcp1-binding surfaces of Mss4 are necessary for an optimal interaction. Interestingly, in the structure model of the Bcp1-Mss4 complex, Bcp1's internal loop, whose first

negatively charged residues are predicted to contact a positively charged surface on the first PIPK subdomain, and the NLS region of Mss4 are not in a configuration that would grant access to their respective importin, thereby providing a plausible explanation for how the Bcp1-Mss4 interaction would prevent the nuclear entry of Mss4. Since Bcp1 binding is predicted to efficiently shield all prominent positively charged surfaces of Mss4 (Supplementary Fig. S9C), it is likely that Bcp1 also ensures the safe transfer of Mss4 to the plasma membrane, a scenario that is fully compatible with the previously reported cell biological and genetic data [22].

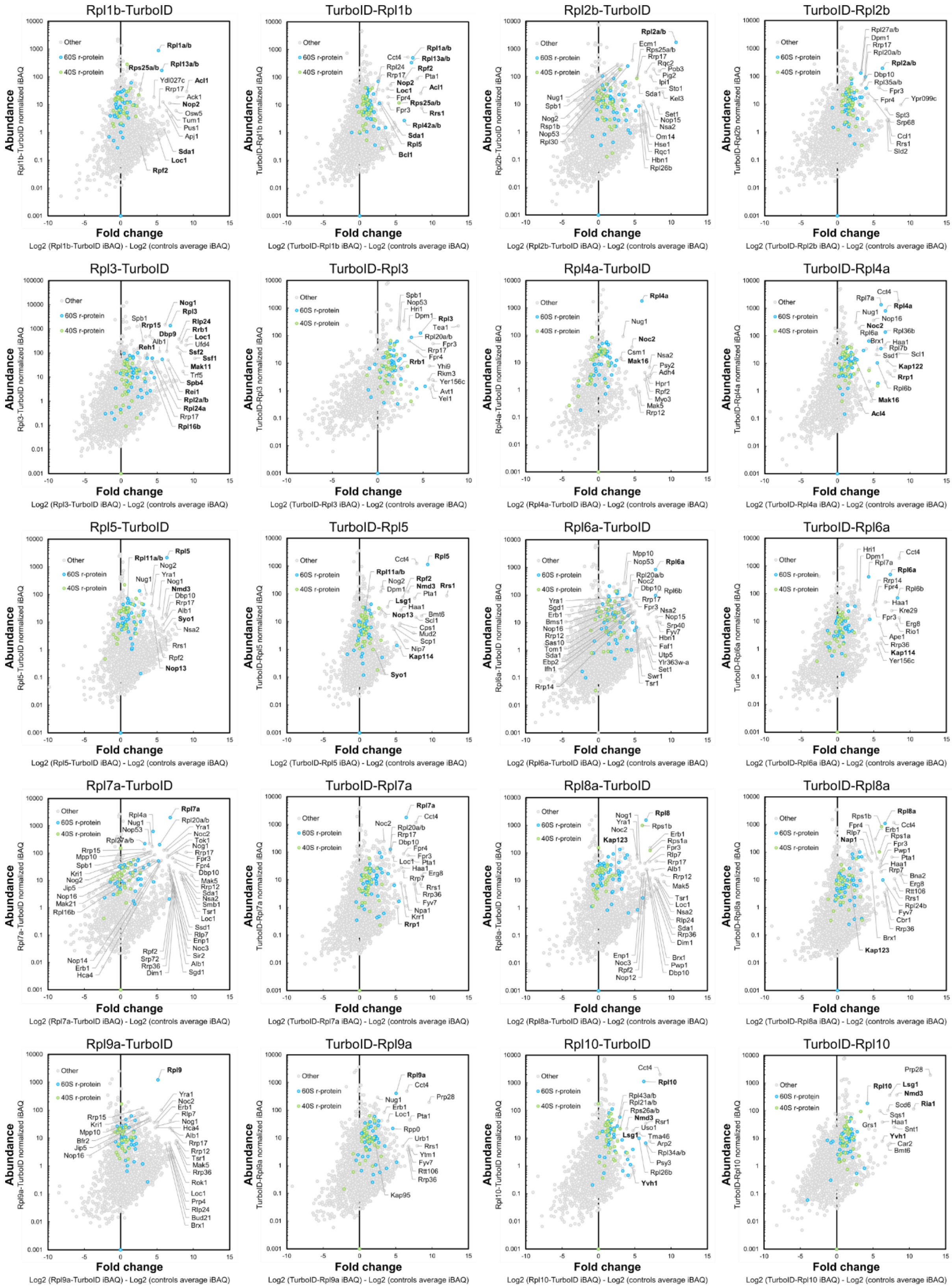
Rpl1b-TurboID
TurboID-Rpl1b
Rpl2b-TurboID
TurboID-Rpl2b
Rpl3-TurboID
TurboID-Rpl3
Rpl4a-TurboID
TurboID-Rpl4a
Rpl5-TurboID
TurboID-Rpl5
Rpl6a-TurboID
TurboID-Rpl6a
Rpl7a-TurboID
TurboID-Rpl7a
Rpl8a-TurboID
TurboID-Rpl8a
Rpl9a-TurboID
TurboID-Rpl9a
Rpl10-TurboID
TurboID-Rpl10
Other
60S r-protein
40S r-protein
Abundance
Fold change
Log2 (Rpl1b-TurboID iBAQ) - Log2 (controls average iBAQ)
Log2 (TurboID-Rpl1b iBAQ) - Log2 (controls average iBAQ)
Log2 (Rpl2b-TurboID iBAQ) - Log2 (controls average iBAQ)
Log2 (TurboID-Rpl2b iBAQ) - Log2 (controls average iBAQ)
Log2 (Rpl3-TurboID iBAQ) - Log2 (controls average iBAQ)
Log2 (TurboID-Rpl3 iBAQ) - Log2 (controls average iBAQ)
Log2 (Rpl4a-TurboID iBAQ) - Log2 (controls average iBAQ)
Log2 (TurboID-Rpl4a iBAQ) - Log2 (controls average iBAQ)
Log2 (Rpl5-TurboID iBAQ) - Log2 (controls average iBAQ)
Log2 (TurboID-Rpl5 iBAQ) - Log2 (controls average iBAQ)
Log2 (Rpl6a-TurboID iBAQ) - Log2 (controls average iBAQ)
Log2 (TurboID-Rpl6a iBAQ) - Log2 (controls average iBAQ)
Log2 (Rpl7a-TurboID iBAQ) - Log2 (controls average iBAQ)
Log2 (TurboID-Rpl7a iBAQ) - Log2 (controls average iBAQ)
Log2 (Rpl8a-TurboID iBAQ) - Log2 (controls average iBAQ)
Log2 (TurboID-Rpl8a iBAQ) - Log2 (controls average iBAQ)
Log2 (Rpl9a-TurboID iBAQ) - Log2 (controls average iBAQ)
Log2 (TurboID-Rpl9a iBAQ) - Log2 (controls average iBAQ)
Log2 (Rpl10-TurboID iBAQ) - Log2 (controls average iBAQ)
Log2 (TurboID-Rpl10 iBAQ) - Log2 (controls average iBAQ)

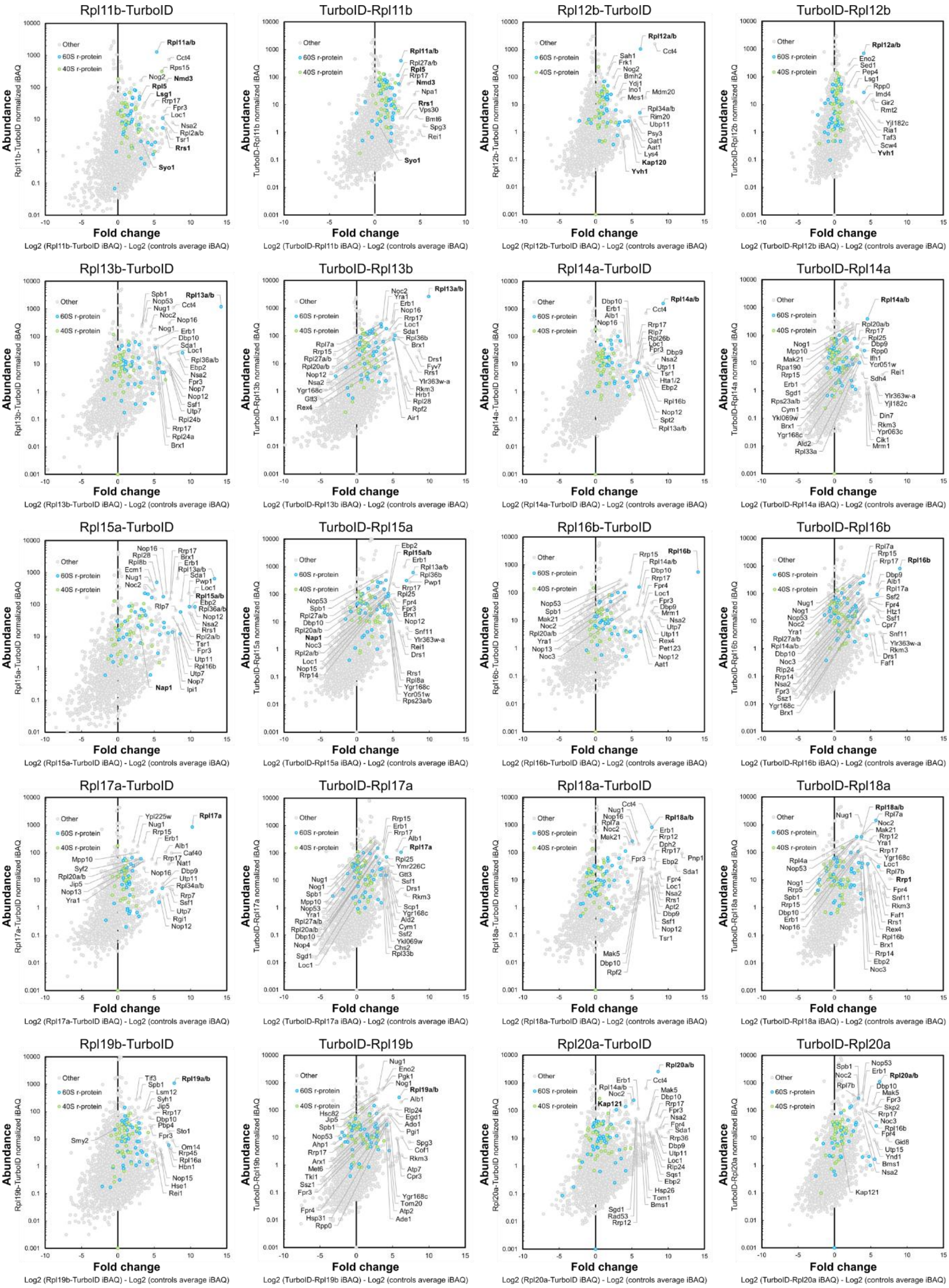
Rpl11b-TurboID
TurboID-Rpl11b
Rpl12b-TurboID
TurboID-Rpl12b
Rpl13b-TurboID
TurboID-Rpl13b
Rpl14a-TurboID
TurboID-Rpl14a
Rpl15a-TurboID
TurboID-Rpl15a
Rpl16b-TurboID
TurboID-Rpl16b
Rpl17a-TurboID
TurboID-Rpl17a
Rpl18a-TurboID
TurboID-Rpl18a
Rpl19b-TurboID
TurboID-Rpl19b
Rpl20a-TurboID
TurboID-Rpl20a
Other
60S r-protein
40S r-protein
Abundance
Fold change
Log2 (Rpl11b-TurboID iBAQ) - Log2 (controls average iBAQ)
Log2 (TurboID-Rpl11b iBAQ) - Log2 (controls average iBAQ)
Log2 (Rpl12b-TurboID iBAQ) - Log2 (controls average iBAQ)
Log2 (TurboID-Rpl12b iBAQ) - Log2 (controls average iBAQ)
Log2 (Rpl13b-TurboID iBAQ) - Log2 (controls average iBAQ)
Log2 (TurboID-Rpl13b iBAQ) - Log2 (controls average iBAQ)
Log2 (Rpl14a-TurboID iBAQ) - Log2 (controls average iBAQ)
Log2 (TurboID-Rpl14a iBAQ) - Log2 (controls average iBAQ)
Log2 (Rpl15a-TurboID iBAQ) - Log2 (controls average iBAQ)
Log2 (TurboID-Rpl15a iBAQ) - Log2 (controls average iBAQ)
Log2 (Rpl16b-TurboID iBAQ) - Log2 (controls average iBAQ)
Log2 (TurboID-Rpl16b iBAQ) - Log2 (controls average iBAQ)
Log2 (Rpl17a-TurboID iBAQ) - Log2 (controls average iBAQ)
Log2 (TurboID-Rpl17a iBAQ) - Log2 (controls average iBAQ)
Log2 (Rpl18a-TurboID iBAQ) - Log2 (controls average iBAQ)
Log2 (TurboID-Rpl18a iBAQ) - Log2 (controls average iBAQ)
Log2 (Rpl19b-TurboID iBAQ) - Log2 (controls average iBAQ)
Log2 (TurboID-Rpl19b iBAQ) - Log2 (controls average iBAQ)
Log2 (Rpl20a-TurboID iBAQ) - Log2 (controls average iBAQ)
Log2 (TurboID-Rpl20a iBAQ) - Log2 (controls average iBAQ)

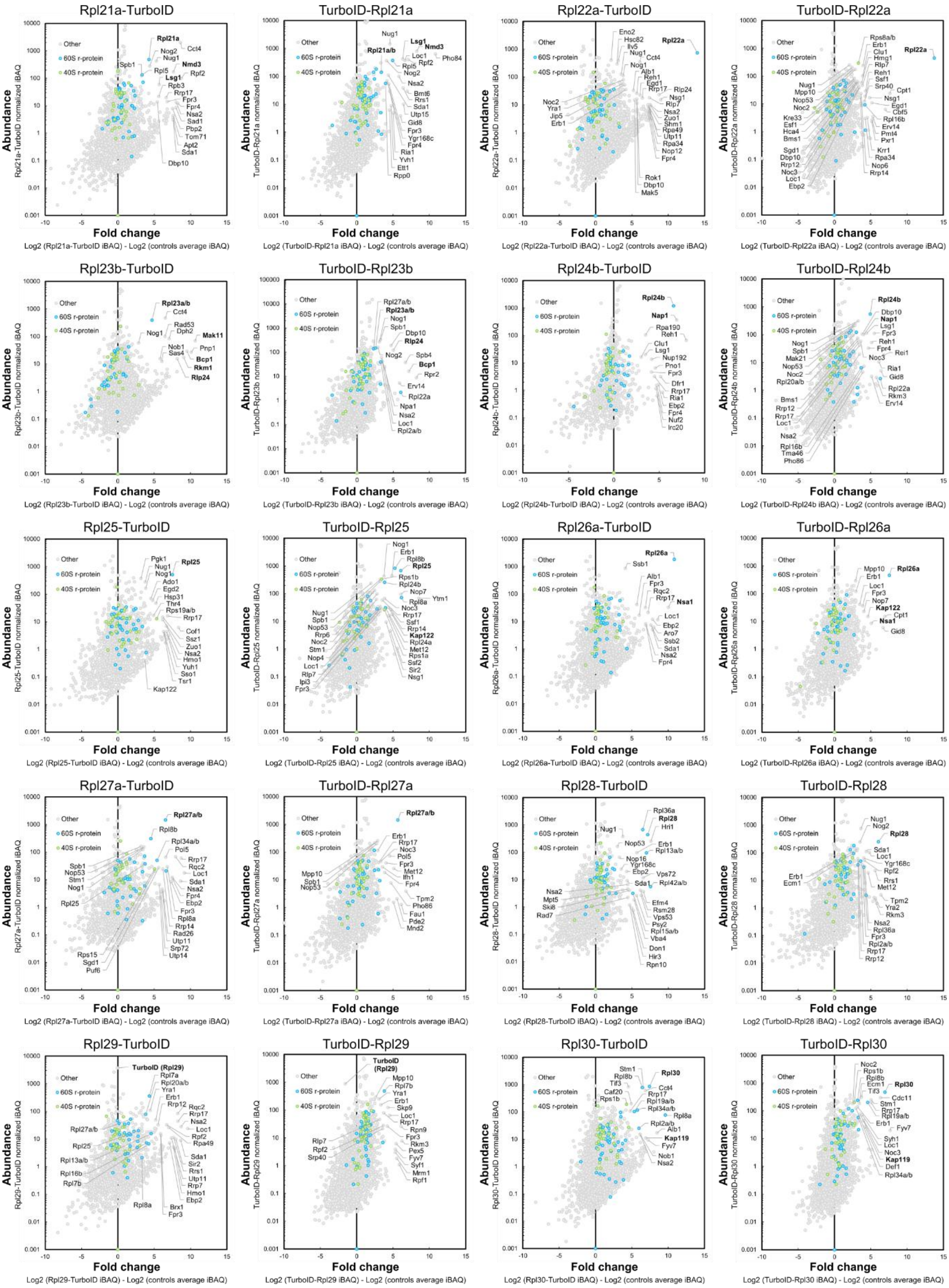
Rpl21a-TurboID
TurboID-Rpl21a
Rpl22a-TurboID
TurboID-Rpl22a
Rpl23b-TurboID
TurboID-Rpl23b
Rpl24b-TurboID
TurboID-Rpl24b
Rpl25-TurboID
TurboID-Rpl25
Rpl26a-TurboID
TurboID-Rpl26a
Rpl27a-TurboID
TurboID-Rpl27a
Rpl28-TurboID
TurboID-Rpl28
Rpl29-TurboID
TurboID-Rpl29
Rpl30-TurboID
TurboID-Rpl30
Other
60S r-protein
40S r-protein
Abundance
Fold change

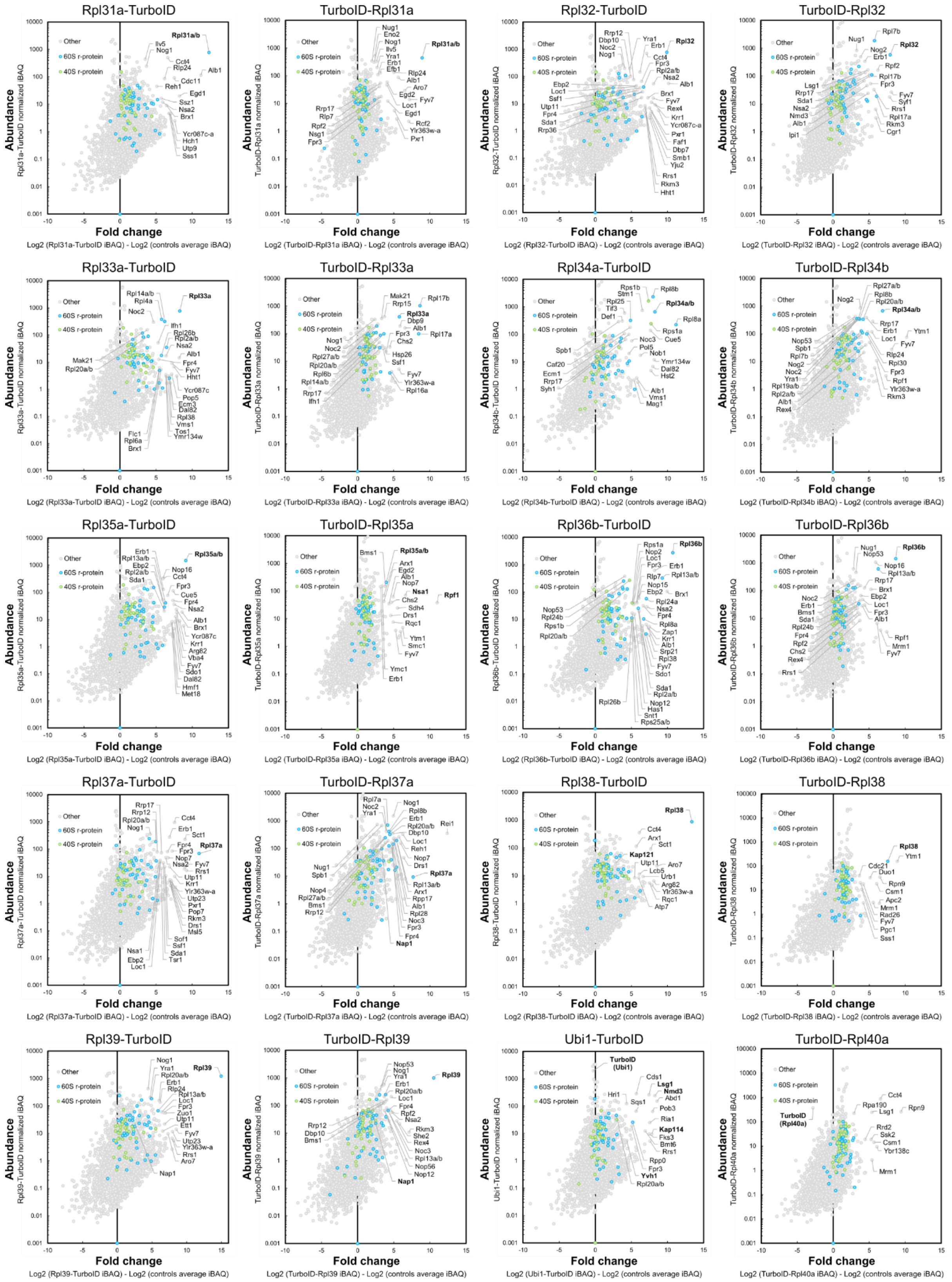
Rpl31a-TurboID
TurboID-Rpl31a
Rpl32-TurboID
TurboID-Rpl32
Rpl33a-TurboID
TurboID-Rpl33a
Rpl34a-TurboID
TurboID-Rpl34b
Rpl35a-TurboID
TurboID-Rpl35a
Rpl36b-TurboID
TurboID-Rpl36b
Rpl37a-TurboID
TurboID-Rpl37a
Rpl38-TurboID
TurboID-Rpl38
Rpl39-TurboID
TurboID-Rpl39
Ubi1-TurboID
TurboID-Rpl40a
Other
60S r-protein
40S r-protein
Abundance
Fold change
Log2 (Rpl31a-TurboID iBAQ) - Log2 (controls average iBAQ)
Log2 (TurboID-Rpl31a iBAQ) - Log2 (controls average iBAQ)
Log2 (Rpl32-TurboID iBAQ) - Log2 (controls average iBAQ)
Log2 (TurboID-Rpl32 iBAQ) - Log2 (controls average iBAQ)
Log2 (Rpl33a-TurboID iBAQ) - Log2 (controls average iBAQ)
Log2 (TurboID-Rpl33a iBAQ) - Log2 (controls average iBAQ)
Log2 (Rpl34b-TurboID iBAQ) - Log2 (controls average iBAQ)
Log2 (TurboID-Rpl34b iBAQ) - Log2 (controls average iBAQ)
Log2 (Rpl35a-TurboID iBAQ) - Log2 (controls average iBAQ)
Log2 (TurboID-Rpl35a iBAQ) - Log2 (controls average iBAQ)
Log2 (Rpl36b-TurboID iBAQ) - Log2 (controls average iBAQ)
Log2 (TurboID-Rpl36b iBAQ) - Log2 (controls average iBAQ)
Log2 (Rpl37a-TurboID iBAQ) - Log2 (controls average iBAQ)
Log2 (TurboID-Rpl37a iBAQ) - Log2 (controls average iBAQ)
Log2 (Rpl38-TurboID iBAQ) - Log2 (controls average iBAQ)
Log2 (TurboID-Rpl38 iBAQ) - Log2 (controls average iBAQ)
Log2 (Rpl39-TurboID iBAQ) - Log2 (controls average iBAQ)
Log2 (TurboID-Rpl39 iBAQ) - Log2 (controls average iBAQ)
Log2 (Ubi1-TurboID iBAQ) - Log2 (controls average iBAQ)
Log2 (TurboID-Rpl40a iBAQ) - Log2 (controls average iBAQ)

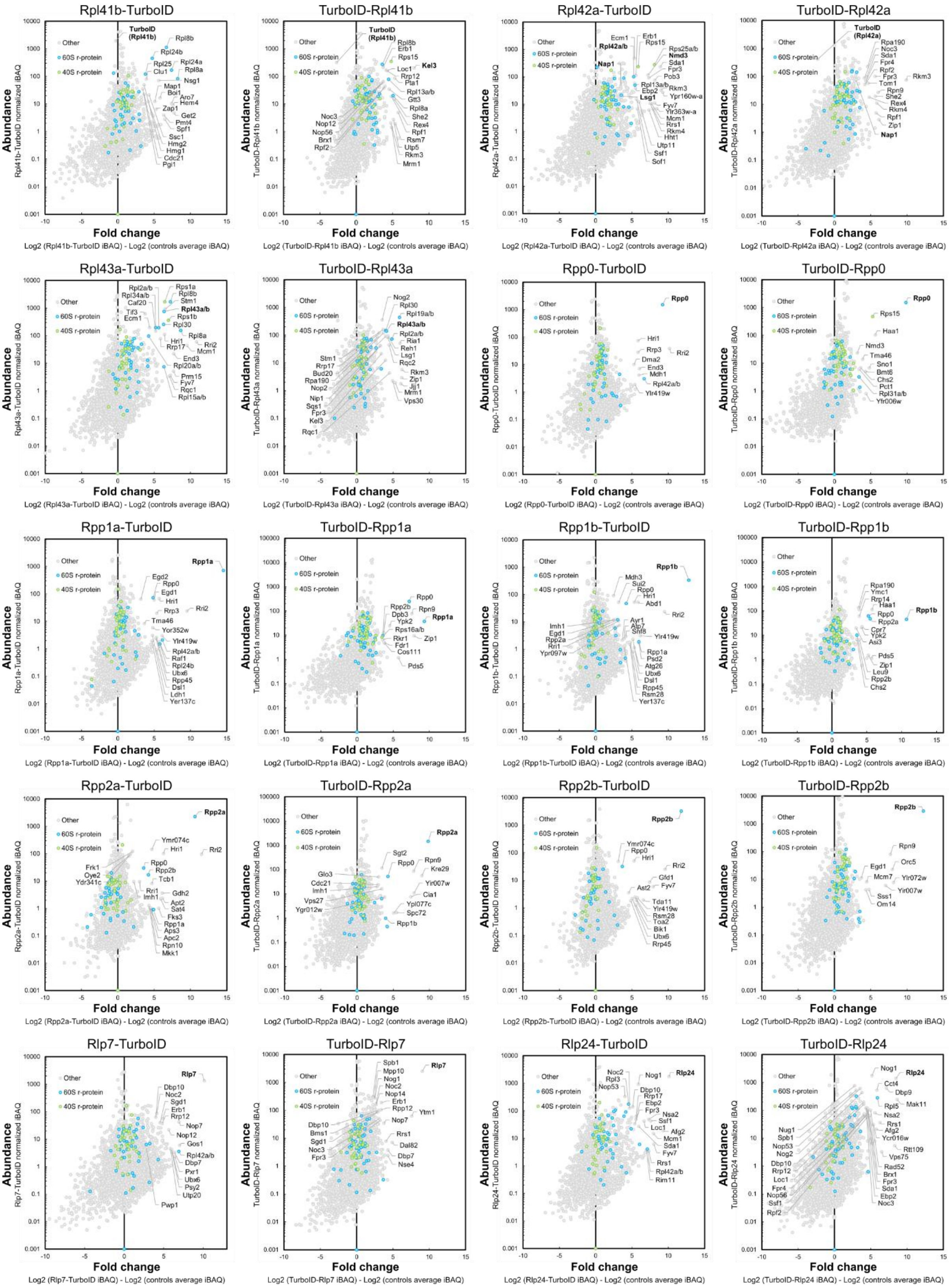
Rpl41b-TurboID
TurboID-Rpl41b
Rpl42a-TurboID
TurboID-Rpl42a
Rpl43a-TurboID
TurboID-Rpl43a
Rpp0-TurboID
TurboID-Rpp0
Rpp1a-TurboID
TurboID-Rpp1a
Rpp1b-TurboID
TurboID-Rpp1b
Rpp2a-TurboID
TurboID-Rpp2a
Rpp2b-TurboID
TurboID-Rpp2b
Rlp7-TurboID
TurboID-Rlp7
Rlp24-TurboID
TurboID-Rlp24
Other
60S r-protein
40S r-protein
Abundance
Fold change

Supplementary Figure S1. The proxiOME of LSU r-proteins. Graphical representation of the TurboID results obtained with all N- and C-terminally TurboID-tagged LSU r-proteins as well as with the ribosomal-like AFs Rlp7 and Rlp24. The employed bait protein is indicated above each graph. The bait proteins, enriched DCs and importins, and selected enriched r-proteins and AFs are written in bold. Note that in the case of the N- and C-terminally TurboID-tagged Rpl29, Rpl40a/Ubi1, and Rpl41b bait proteins and of TurboID-Rpl42a only the TurboID moiety but not the fused r-protein could be detected in the analysis of non-biotinylated tryptic peptides, which were used for the identification and quantification of proteins. In these cases, the abundance and relative enrichment of the fused TurboID moiety is indicated in the graphs.

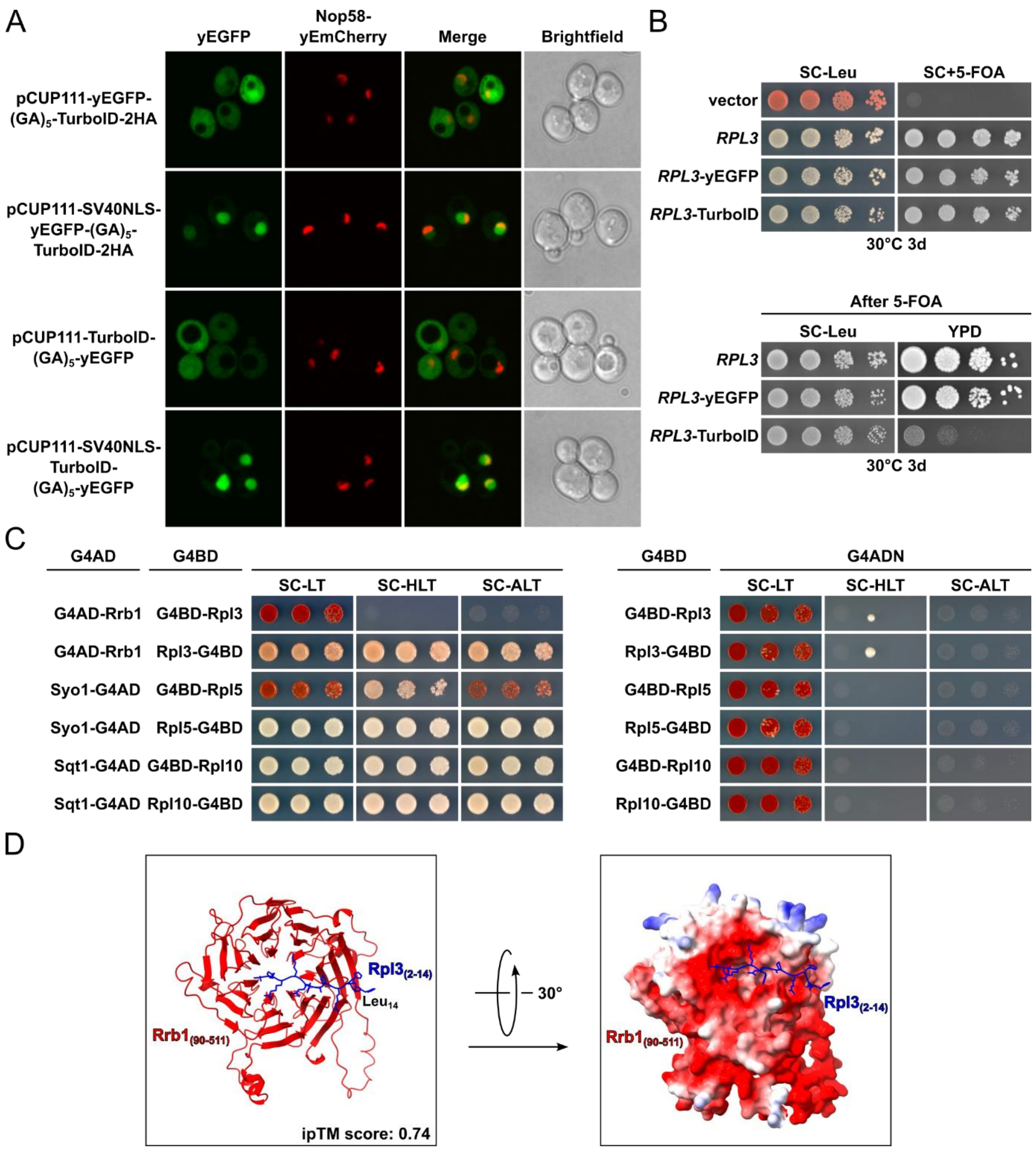

Supplementary Figure S2. (A) Localization of the four TurboID-tagged GFP control proteins, expressed for 1 h in the presence of 0.1 mM $CuSO_4$ under the control of the *CUP1* promoter from plasmid, was assessed by fluorescence microscopy in cells expressing the nucleolar marker protein Nop58-yEmCherry from the genomic locus that were grown in SC-Leu medium at 30ºC. **(B)** Evaluation of the functionality of C-terminally TurboID-tagged Rpl3. Empty vector (YCplac111) and plasmids harbouring *RPL3*, *RPL3*-yEGFP, or *RPL3*-TurboID, expressed under the transcriptional control of the *RPL3* promoter, were transformed into an *RPL3* shuffle strain. Transformants were restreaked on SC-Leu plates and cells were then spotted in 10-fold serial dilution steps onto SC-Leu plates and SC plates containing 5-fluoroorotic acid (5-FOA), which were incubated for 3 days at 30°C. After plasmid shuffling, cells were restreaked on SC-Leu plates and then spotted in 10-fold serial dilution steps onto SC-Leu and YPD plates. **(C)** Y2H interaction assays between the r-proteins Rpl3, Rpl5, and Rpl10 (tagged at their N- or C-termini with the G4BD) and their respective G4AD-tagged DC Rrb1, Syo1,

and Sqt1. **(D)** AlphaFold3 model of the Rrb1-Rpl3 complex. The prediction was done with full-length Rrb1 and Rpl3 lacking its N-terminal methionine as input, but, for clarity, only residues 90-511 of Rrb1 (red) and residues 2-14 of Rpl3 (blue) are shown in the cartoon representation (left) and the representation depicting the electrostatic surface potential of Rrb1 (right).

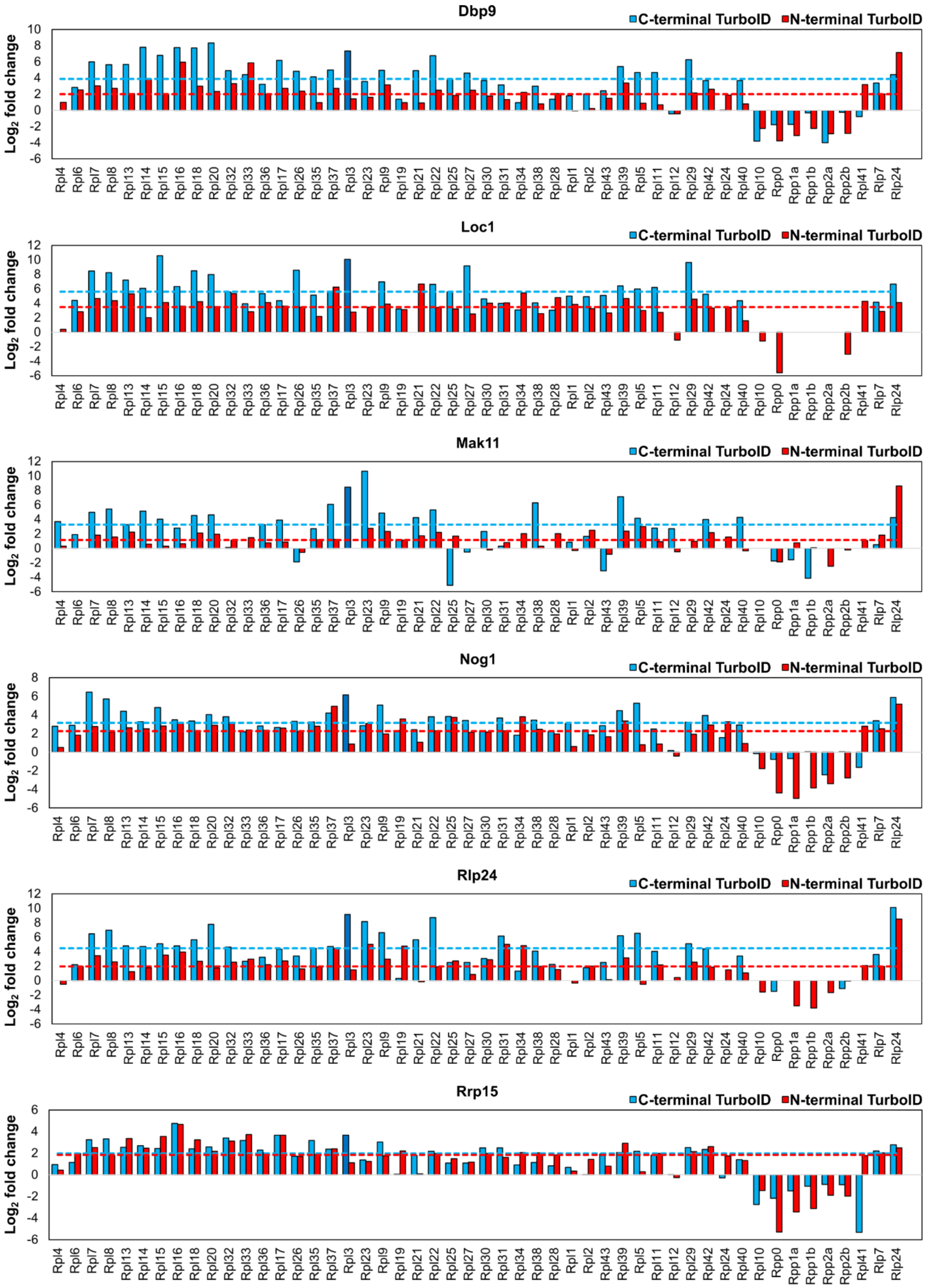

Dbp9
Loc1
Mak11
Nog1
Rlp24
Rrp15
C-terminal TurboID
N-terminal TurboID
Log$_2$ fold change
Rpl4
Rpl6
Rpl7
Rpl8
Rpl13
Rpl14
Rpl15
Rpl16
Rpl18
Rpl20
Rpl32
Rpl33
Rpl36
Rpl17
Rpl26
Rpl35
Rpl37
Rpl3
Rpl23
Rpl9
Rpl19
Rpl21
Rpl22
Rpl25
Rpl27
Rpl30
Rpl31
Rpl34
Rpl38
Rpl28
Rpl1
Rpl2
Rpl43
Rpl39
Rpl5
Rpl11
Rpl12
Rpl29
Rpl42
Rpl24
Rpl40
Rpl10
Rpp0
Rpp1a
Rpp1b
Rpp2a
Rpp2b
Rpl41
Rlp7
Rlp24

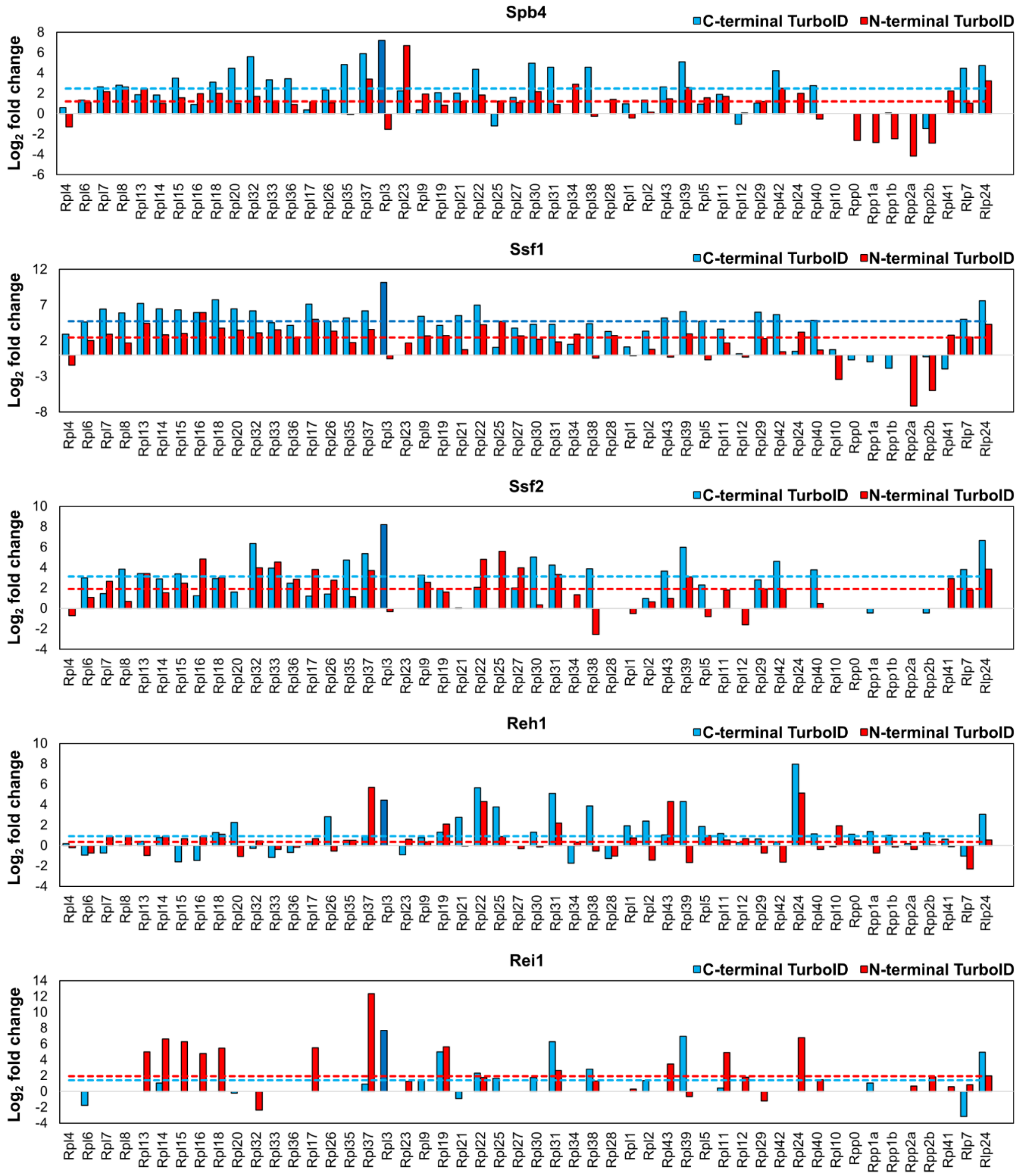

Supplementary Figure S3. Bar graphs showing the log2 fold enrichment of AFs, which were enriched in the TurboID assays with the Rpl3-TurboID bait (see Fig. 1B), in the TurboID assays of all N- (red bars) and C-terminally (blue bars) TurboID-tagged LSU r-proteins as well as the ones of Rlp7 and Rlp24. In the case of the Rpl3-TurboID bait (dark blue bars) and the TurboID-Rpl1 bait, their log2 fold enrichment corresponds to the average of the eight or, respectively, six experimental replicates. Dashed lines indicate the median log2 fold enrichment of an AF across all shown TurboID assays. The order of the LSU r-proteins (from left to right) is according to their first visualization in structures of early nucleolar to cytoplasmic pre-60S particles.

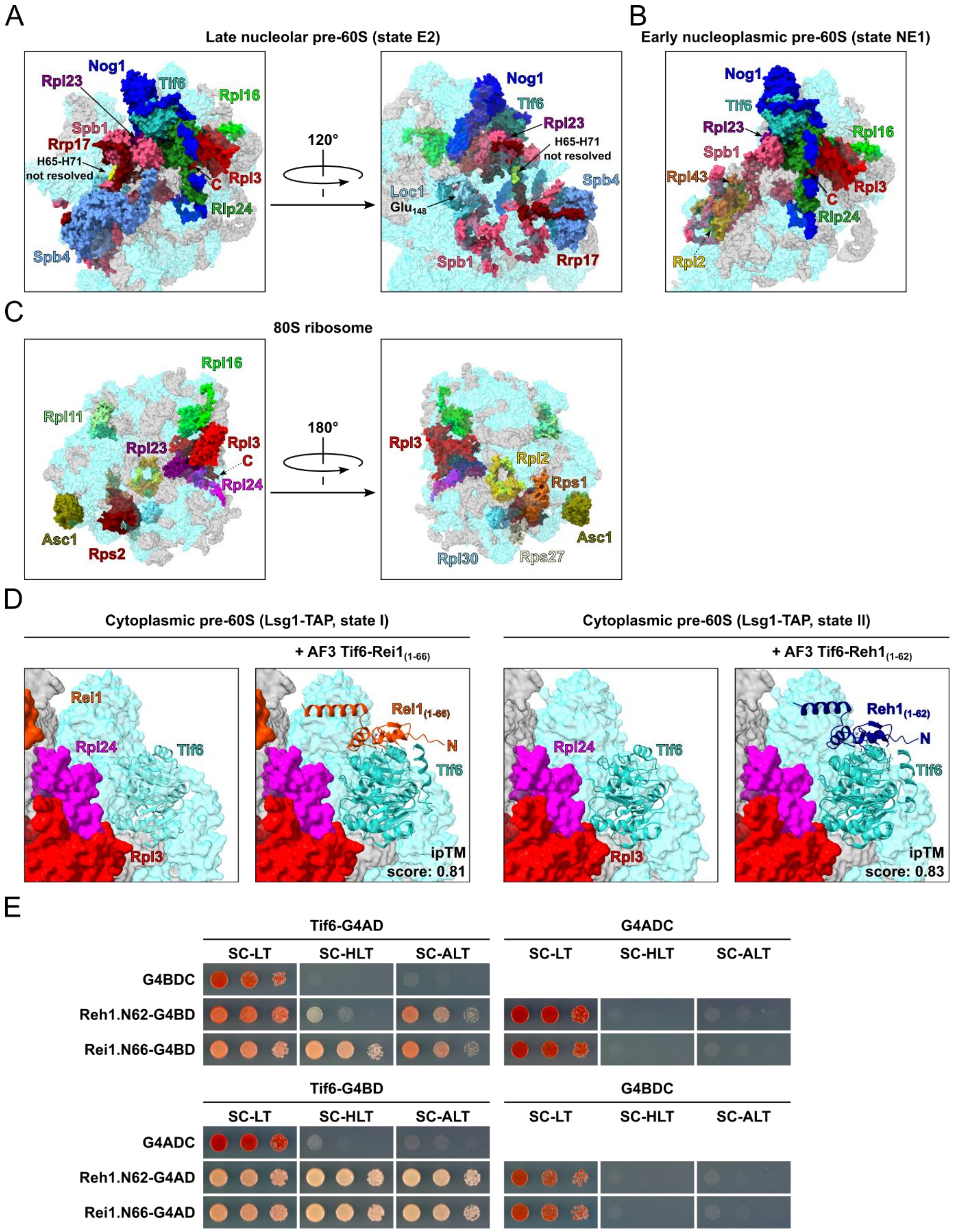

Supplementary Figure S4. (A-C) Location of Rpl3 and its neighbouring proteins on: **(A)** the late nucleolar state E2 pre-60S particle (PDB: 7NAC [8]), **(B)** the early nucleoplasmic state NE1 pre-60S particle (PDB: 7U0H [8]), and **(C)** the 80S ribosome (PDB 4V88 [11]). The indicated proteins have been highlighted in different colours; other r-proteins and AFs are coloured in light cyan and (pre-)rRNAs in light grey. **(D)** Superposition of the AlphaFold3 model of the Tif6-Rei1.N66 (left) and Tif6-Reh1.N62 (right) complex onto Tif6 (shown in cartoon representation) within the cytoplasmic Lsg1-TAP state I (left; PDB: 6RZZ) or Lsg1-TAP state II (right; PDB: 6S05) pre-60S particles [13], in which

either parts of Rei1 or Reh1 can be visualized. Rei1 is coloured in orange and Reh1 in dark blue. **(E)** Y2H interaction assays between Tif6 and Reh1.N62 (residues 1-62) or Rei1.N66 (residues 1-66). Note that multicopy plasmids were used for these Y2H assays.

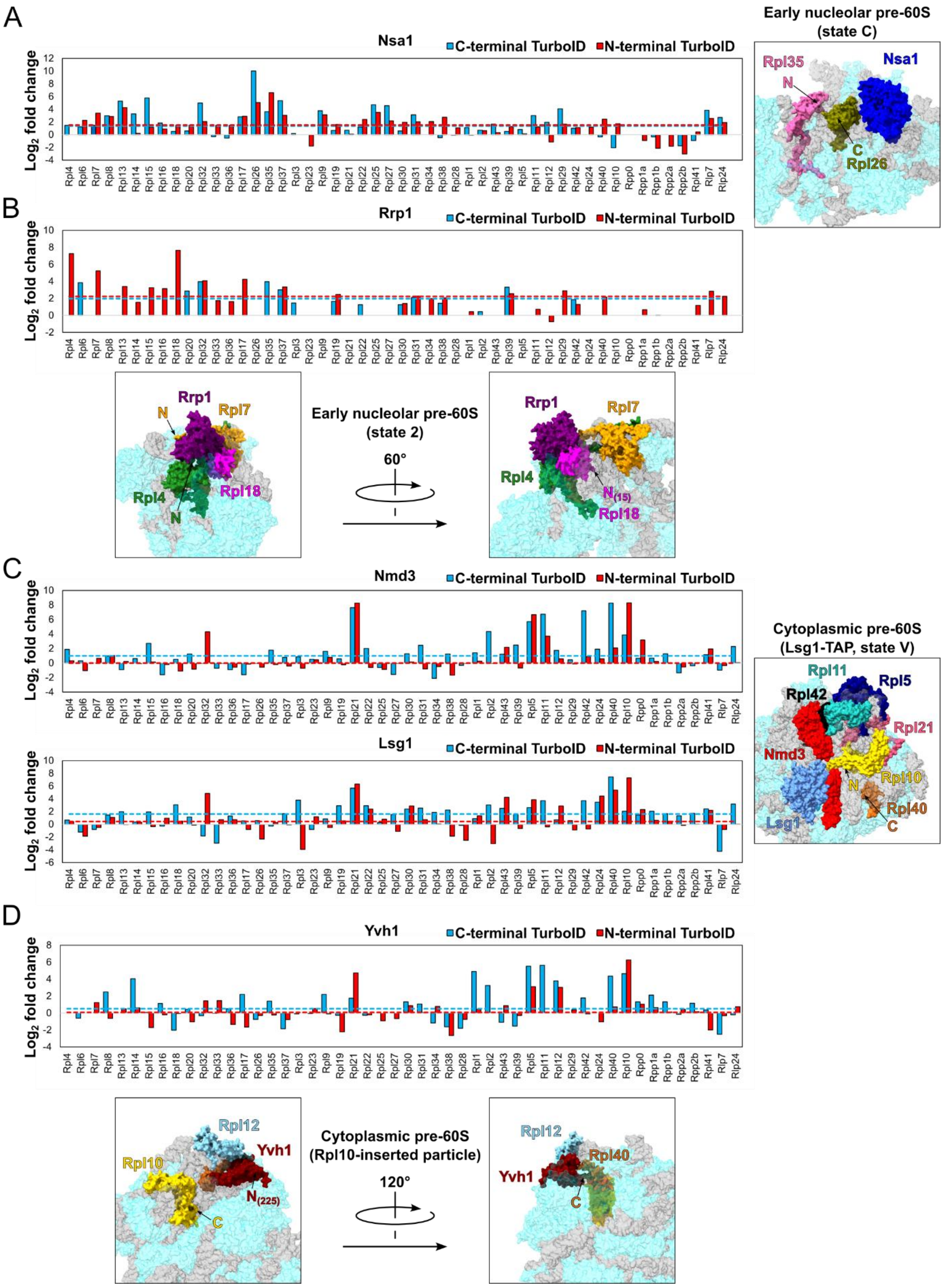

Supplementary Figure S5. (A-D) Bar graphs showing the log2 fold enrichment of Nsa1 **(A)**, Rrp1 **(B)**, Nmd3 and Lsg1 **(C)**, and Yvh1 **(D)** in the TurboID assays of all N- (red bars) and C-terminally (blue bars) TurboID-tagged LSU r-proteins as well as the ones of Rlp7 and Rlp24. In the case of the Rpl3-TurboID bait (dark blue bars) and the TurboID-Rpl1 bait, their log2 fold enrichment corresponds to the

average of the eight or, respectively, six experimental replicates. Dashed lines indicate the median log2 fold enrichment of an AF across all shown TurboID assays. The order of the LSU r-proteins (from left to right) is according to their first visualization in structures of early nucleolar to cytoplasmic pre-60S particles. The right or lower panels show the location and neighbouring proteins of: **(A)** Nsa1 on the early nucleolar state C pre-60S particle (PDB: 6EM1 [2]), **(B)** Rrp1 on the early nucleolar state 2 pre-60S particle (PDB: 6C0F [3]), **(C)** Nmd3 and Lsg1 on a cytoplasmic pre-60S intermediate (Lsg1-TAP, state V; PDB: 6RI5 [13]), **(D)** Yvh1 on the cytoplasmic Rpl10-inserted pre-60S particle (PDB: 6N8O [14]). The indicated proteins have been highlighted in different colours; other r-proteins and AFs are coloured in light cyan and (pre-)rRNAs in light grey.

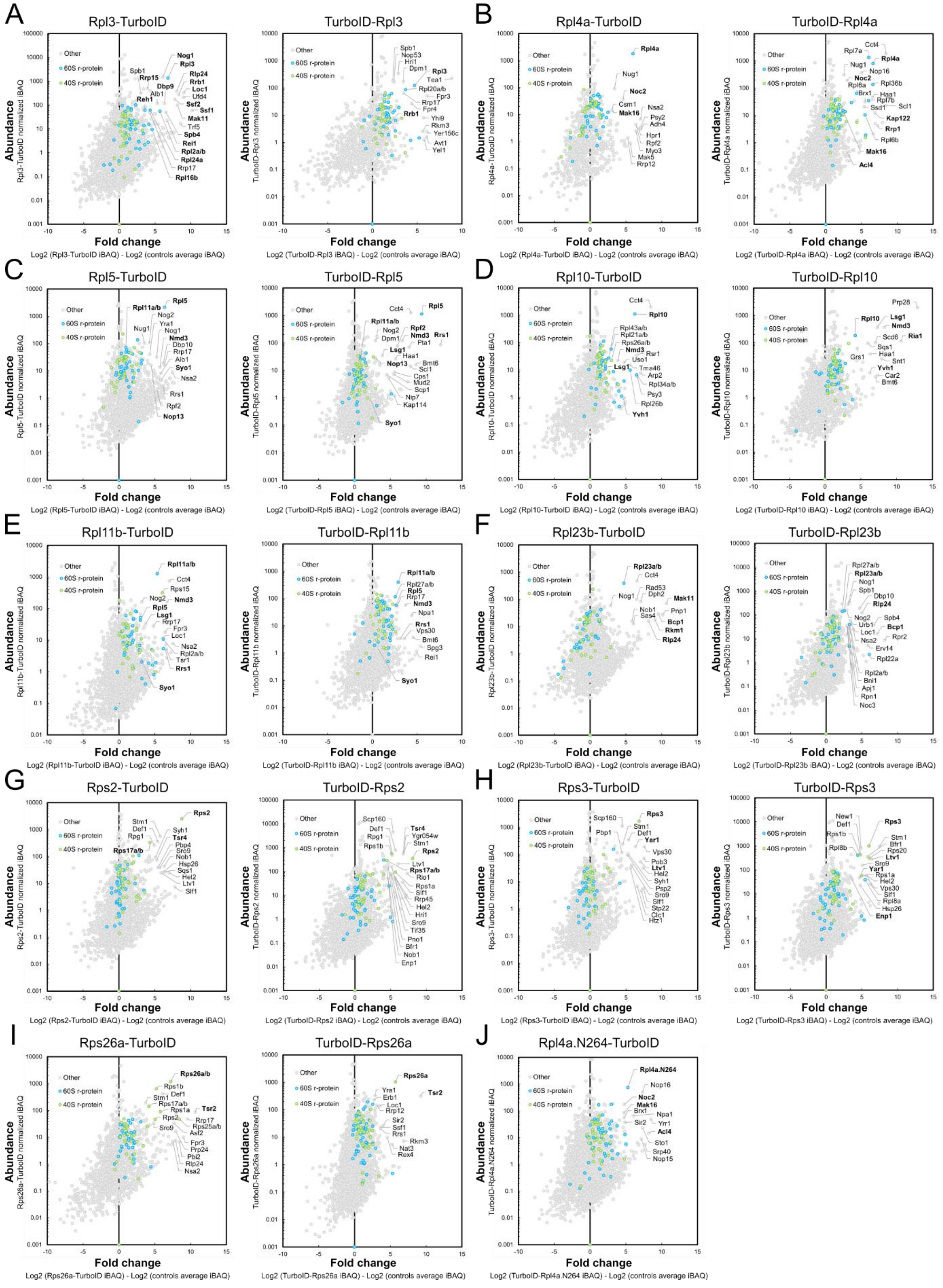

Supplementary Figure S6. Identification of the known DCs in the proxiOMEs of their r-protein clients. (A-I) Graphical representation of the TurboID results obtained with N- and C-terminally TurboID-tagged Rpl3 **(A)**, Rpl4a **(B)**, Rpl5 **(C)**, Rpl10 **(D)**, Rpl11b **(E)**, Rpl23b **(F)**, Rps2 **(G)**, Rps3 **(H)**, and Rps26a **(I)**. **(J)** TurboID result obtained with the C-terminally TurboID-tagged Rpl4 variant

lacking the C-terminal extension (Rpl4a.N264). The employed bait r-protein is indicated above each graph, and the bait r-protein, its known DC, and selected enriched proteins are written in bold.

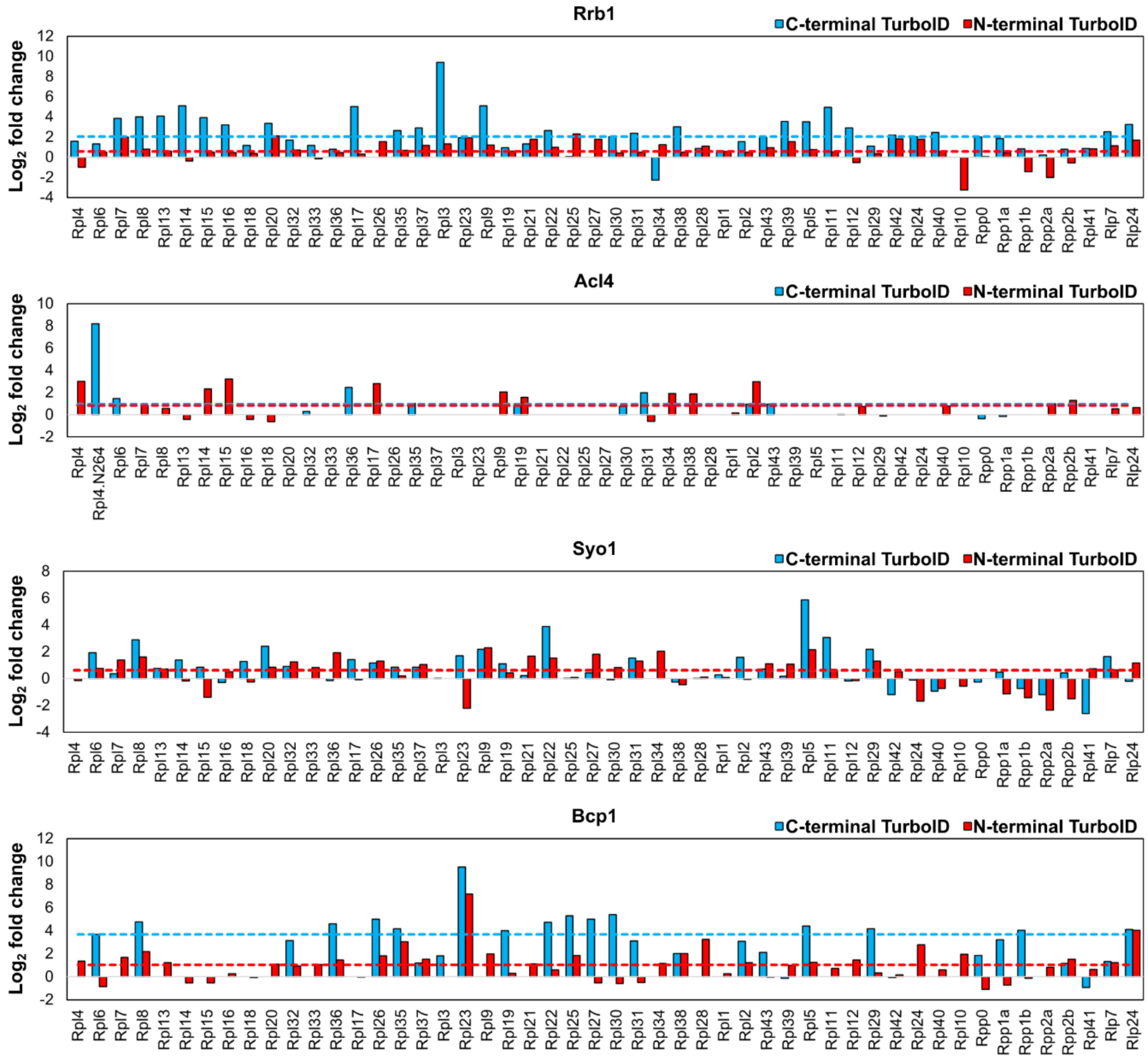

Supplementary Figure S7. The DCs Rrb1, Acl4, Syo1, and Bcp1 are specifically enriched in the TurboID assays of their r-protein client. Bar graphs showing the log2 fold enrichment of Rrb1, Acl4, Syo1, and Bcp1 in the TurboID assays of all N- (red bars) and C-terminally (blue bars) TurboID-tagged LSU r-proteins as well as the ones of Rlp7 and Rlp24. In the case of the Rpl3-TurboID bait and the TurboID-Rpl1 bait, their log2 fold enrichment corresponds to the average of the eight or, respectively, six experimental replicates. Dashed lines indicate the median log2 fold enrichment of an AF across all shown TurboID assays. The order of the LSU r-proteins (from left to right) is according to their first visualization in structures of early nucleolar to cytoplasmic pre-60S particles.

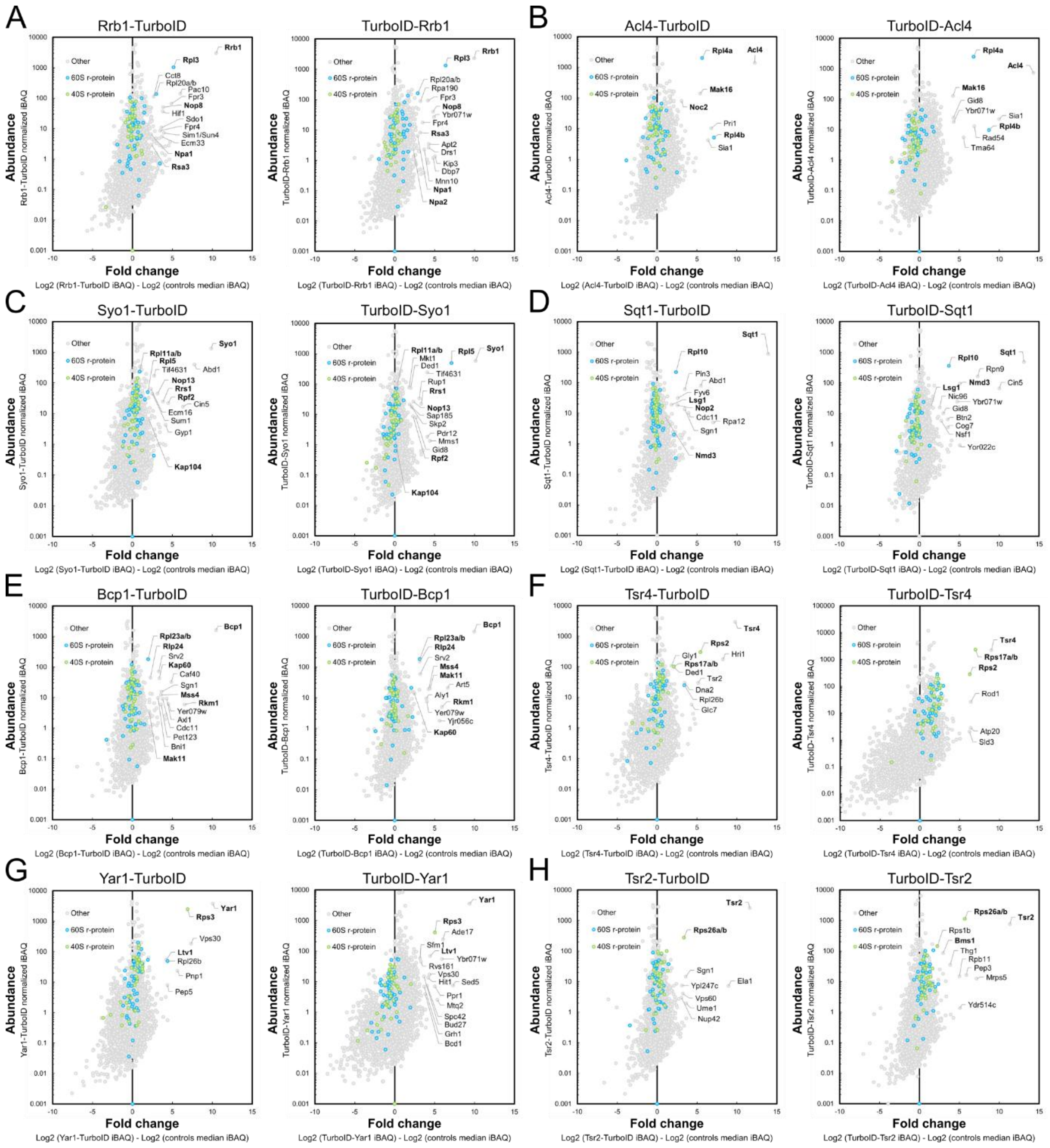

Supplementary Figure S8. Identification of the known r-protein clients in the proxiOMEs of their DCs. (A-H) Graphical representation of the TurboID results obtained with N- and C-terminally TurboID-tagged Rrb1 **(A)**, Acl4 **(B)**, Syo1 **(C)**, Sqt1 **(D)**, Bcp1 **(E)**, Tsr4 **(F)**, Yar1 **(G)**, and Tsr2 **(H)**. The abundance of each detected protein is displayed as its normalized intensity-based absolute quantification (iBAQ) value on the vertical axis, and its relative enrichment (fold change) on the horizontal axis as the log2 fold change of its normalized iBAQ abundance between the bait sample and the median of the remaining DC TurboIDs and the two respective control TurboIDs. The employed bait DC is indicated above each graph, and the bait DC, its known r-protein client(s), and selected enriched proteins are written in bold.

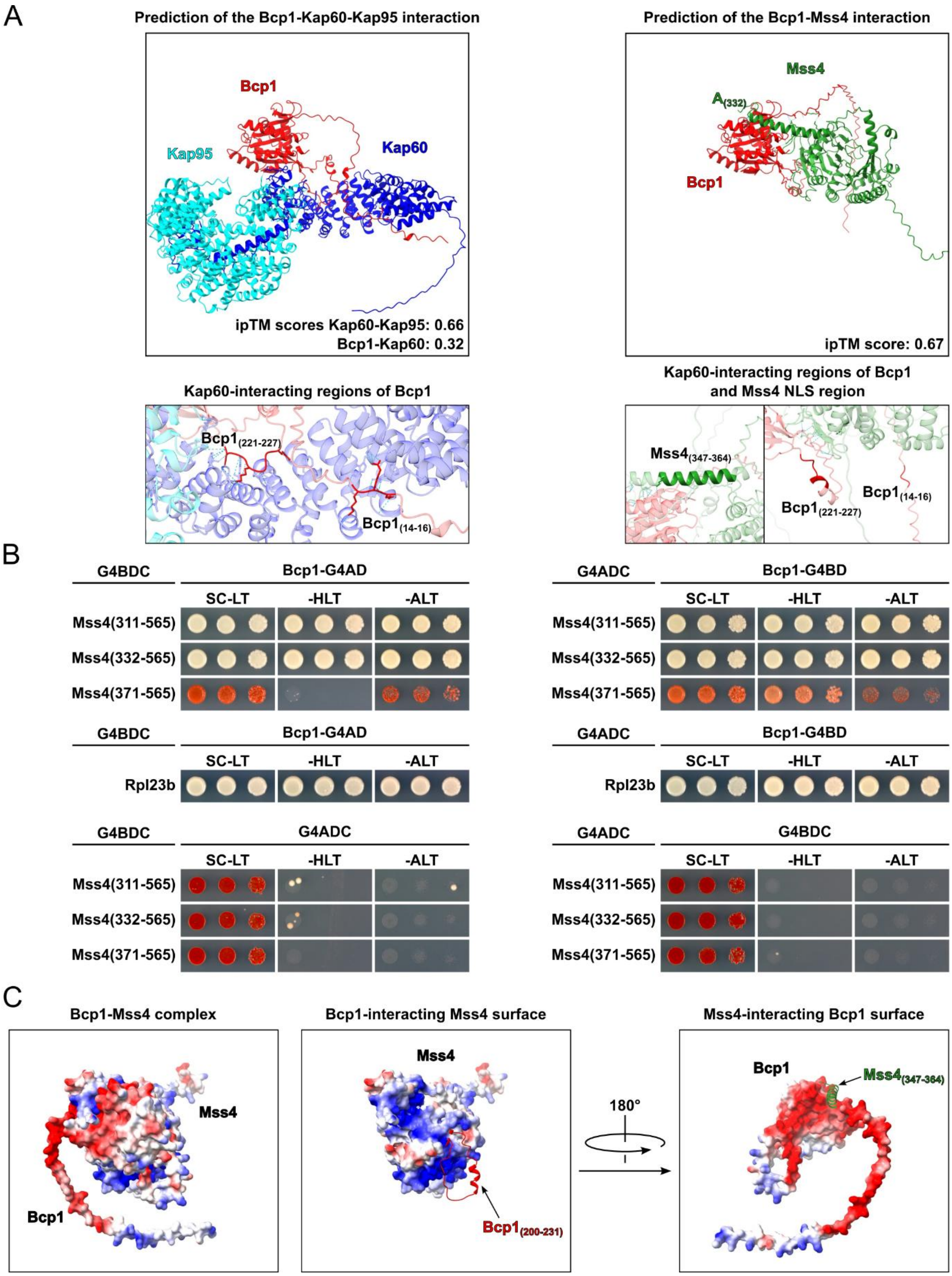

Supplementary Figure S9. Bcp1 shields all prominent positively charged surfaces of Mss4. (A) Cartoon representation of the AlphaFold3 model of the Bcp1-Kap60-Kap95 complex (upper left) and the Bcp1-Mss4 complex (upper right; for clarity of the representation, residues 1-331 of Mss4 have been hidden). The ipTM scores of the individual binary interactions are indicated. Close-up view highlighting the Kap60-interacting residues (14-16 and 221-227; dark red) of Bcp1 in contact with

Kap60 (lower left) or in the Bcp1-Mss4 complex (lower right). The location of the functional NLS region of Mss4 (residues 347-364; [22]) within the predicted Bcp1-Mss4 complex is highlighted in dark green. **(B)** Y2H interaction assays between Bcp1 and Rpl23 (positive control) or the indicated Mss4 segments. **(C)** Predicted electrostatic surface potential of the Bcp1-Mss4 complex (AlphaFold3 model; left), the Bcp1-interacting surface of Mss4 with residues 200-231 of Bcp1 in cartoon representation (middle), and the Mss4-interacting surface of Bcp1 with residues 347-364 of Mss4 in cartoon representation (right), revealing the respective negatively (Bcp1) or positively (Mss4) charged interaction interfaces.

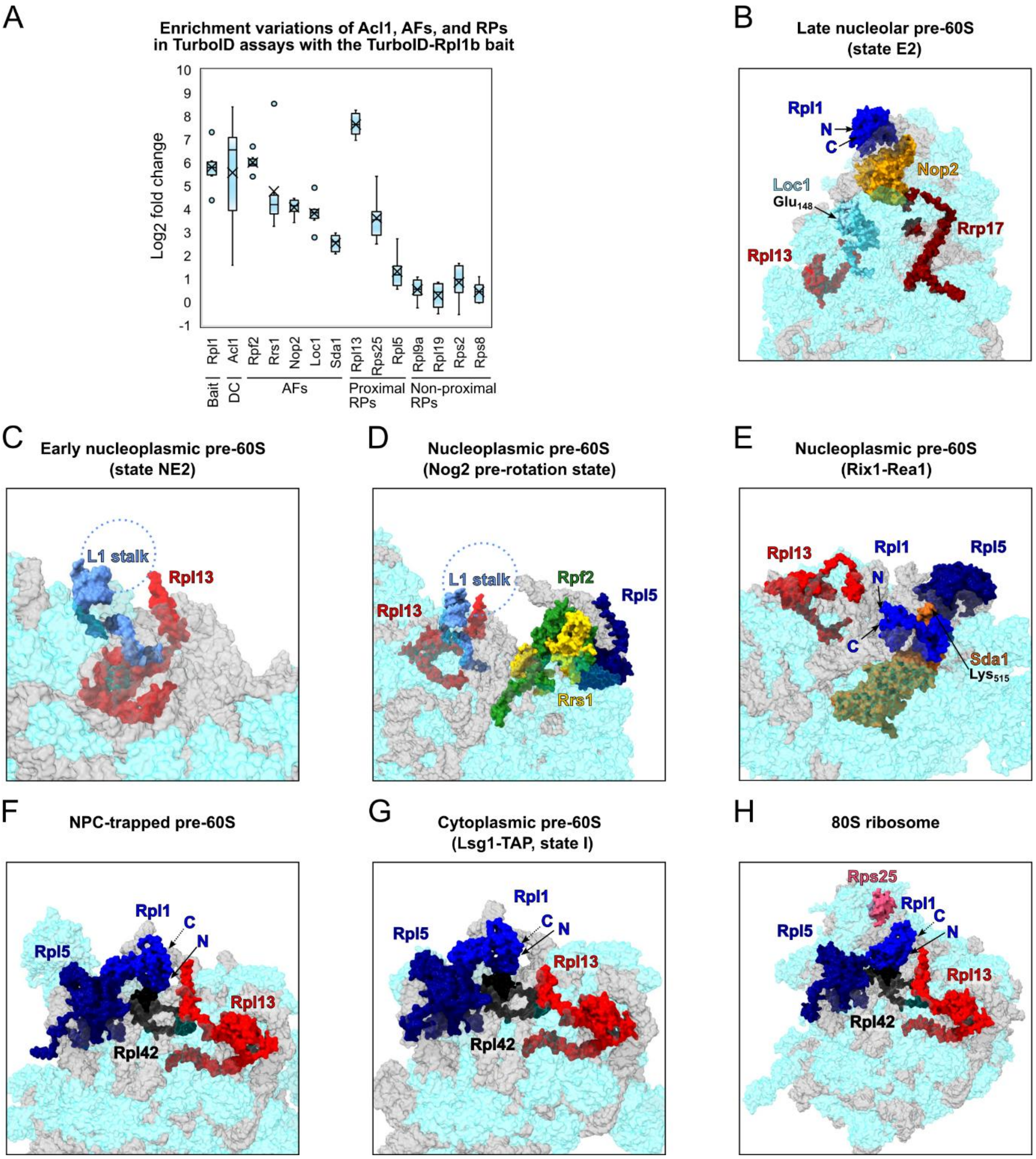

Supplementary Figure S10. ProxiOME of Rpl1. (A) Representation of the variation of the enrichment of the Rpl1 bait, its DC Acl1, proximal AFs, and abundantly detected r-proteins between the experimental replicates (n=6); r-proteins that are close to or far away from Rpl1, as indicated by available structures of pre-60S particles, mature 60S subunits, and 80S ribosomes, are referred to as proximal or non-proximal RPs. The variation of the enrichment is represented by boxes highlighting the interquartile range of each data set, while the whiskers indicate the minimal and maximal limits of the distribution; outliers are shown as dots, the median by a line, and the average by a cross. **(B-H)** Location of Rpl1 or the L1 stalk rRNA and their neighbouring proteins on: **(B)** the late nucleolar state E2 pre-60S particle (PDB: 7NAC [8]), **(C)** the early nucleoplasmic state NE2 pre-60S particle (PDB: 6YLY [10]), **(D)** the nucleoplasmic pre-60S intermediate of the Nog2-containing pre-rotation state (PDB: 7UOO [23]), **(E)** the nucleoplasmic Rix1-Rea1 pre-60S particle (PDB: 6YLH [10]), **(F)** the NPC-trapped pre-60S particle (PDB: 8HFR [24]), **(G)** the cytoplasmic Lsg1-TAP state I pre-60S

particle (PDB: 6RZZ [13]), and **(H)** the 80S ribosome (with the Rpl1-bound L1 stalk rRNA added from the PDB 4V7R 80S structure [25] and superposed on the PDB 4V88 80S structure [11]). The indicated proteins have been highlighted in different colours; other r-proteins and AFs are coloured in light cyan and (pre-)rRNAs in light grey.

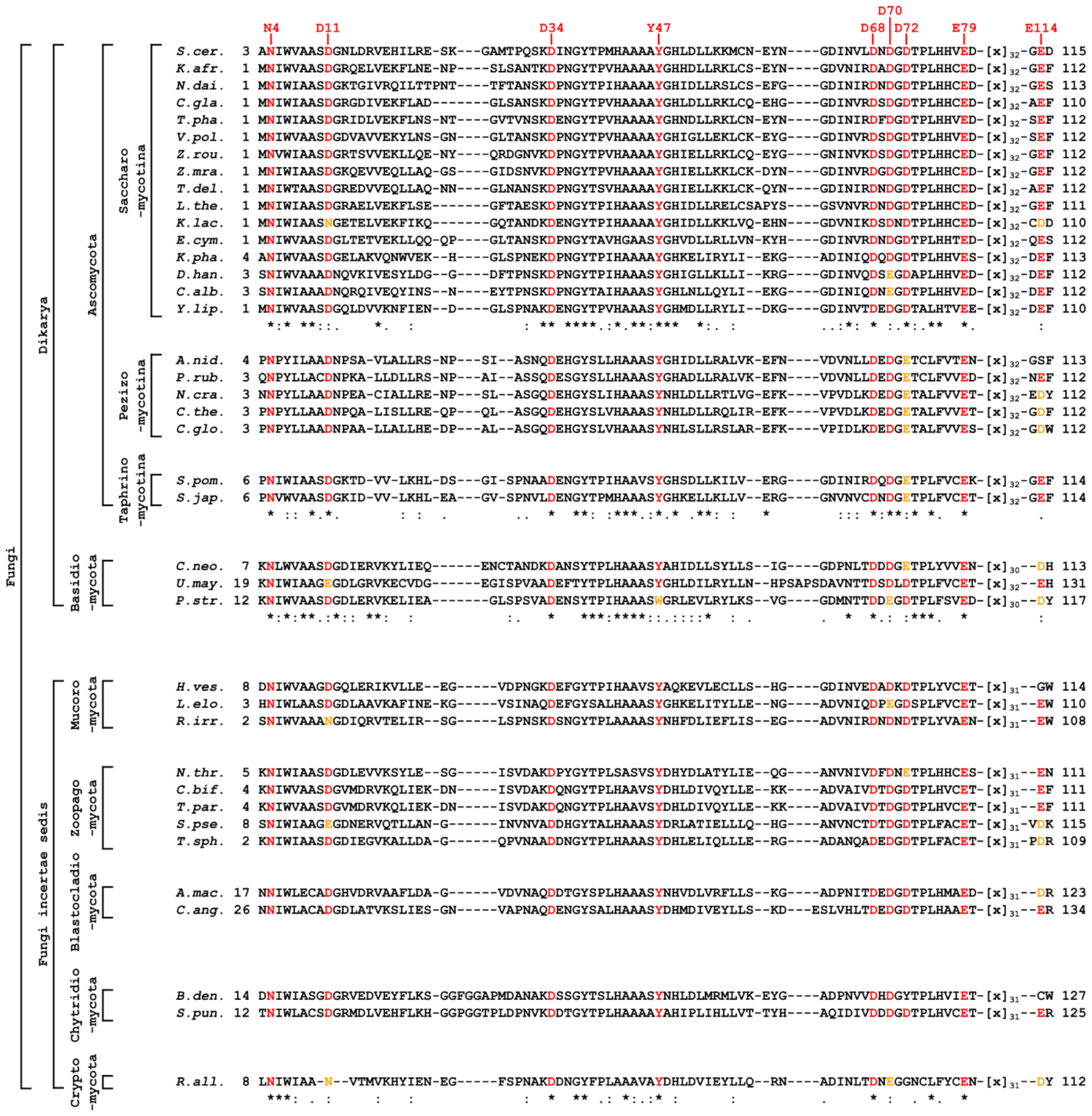

Supplementary Figure S11. Acl1 and its identified Rpl1-interacting residues are conserved within the Fungi kingdom. The amino acid sequence of *S. cerevisiae* Acl1 (*S.cer.*, UniProt: P25631) was aligned with the amino acid sequences of selected potential orthologous proteins, identified with the BLAST function of the UniProtKB [26], belonging to a specific subkingdom, phylum, or subphylum of the Fungi kingdom (see Materials and Methods). For clarity, only the alignment parts covering most of the N-terminal ankyrin repeat domain (residues 3-80 and 113-115 of *S. cerevisiae* Acl1) are shown, and interaction-mediating residues of *S. cerevisiae* Acl1 (indicated on top) that are conserved or semi-conserved are coloured in red or orange. Kingdom, subkingdom, phylum, and subphylum names are indicated. Species names are abbreviated as follows (with the UniProtKB or UniParc number of the respective protein referenced between parentheses): *Kazachstania africana*, *K.afr.* (H2B114); *Naumovozyma dairenensis*, *N.dai.* (G0WFQ9); *Candida glabrata*, *C.gla.* (Q6FWT6); *Tetrapisispora phaffii*, *T.pha.* (G8BT41); *Vanderwaltozyma polyspora*, *V.pol.* (A7TG26); *Zygosaccharomyces rouxii*, *Z.rou.* (C5DVQ9); *Zygotorulaspora mrakii*, *Z.mra.* (A0A7H9AWJ3); *Torulaspora delbrueckii*, *T.del.* (G8ZN72); *Lachancea thermotolerans*, *L.the.* (C5DBY1); *Kluyveromyces lactis*, *K.lac.* (Q6CTY4); *Eremothecium cymbalariae*, *E.cym.* (G8JPK3); *Komagataella phaffii*, *K.pha.* (C4R8E0);

Debaryomyces hansenii, *D.han.* (Q6BK78); *Candida albicans*, *C.alb.* (A0A1D8PHS4); *Yarrowia lipolytica*, *Y.lip.* (Q6C254); *Aspergillus nidulans*, *A.nid.* (Q5BDT5); *Penicillium rubens*, *P.rub.* (B6H7C4); *Neurospora crassa*, *N.cra.* (Q7RZ65); *Chaetomium thermophilum*, *C.the.* (G0S6G3); *Chaetomium globosum*, *C.glo.* (Q2H182), *Schizosaccharomyces pombe*, *S.pom.* (Q9UU77); *Schizosaccharomyces japonicus*, *S.jap.* (B6JYY9); *Cryptococcus neoformans*, *C.neo.* (Q5KM81); *Ustilago maydis*, *U.may.* (A0A0D1E9K4); *Puccinia striiformis*, *P.str.* (A0A2S4WKI7); *Hesseltinella vesiculosa*, *H.ves.* (A0A1X2GXH0); *Linnemannia elongata*, *L.elo.* (A0A197K2N3); *Rhizophagus irregularis*, *R.irr.* (U9U5S0); *Neoconidiobolus thromboides*, *N.thr.* (UPI002211511C); *Coemansia biformis*, *C.bif.* (A0A9W8CY32); *Tieghemiomyces parasiticus*, *T.par.* (A0A9W8ADQ8); *Syncephalis pseudoplumigaleata*, *S.pse.* (A0A4P9YSW2); *Thamnocephalis sphaerospora*, *T.sph.* (A0A4P9XN32); *Allomyces macrogynus*, *A.mac.* (A0A0L0S8J9); *Catenaria anguillulae*, *C.ang.* (A0A1Y2HCN5); *Batrachochytrium dendrobatidis*, *B.den.* (A0A177W6V9); *Spizellomyces punctatus*, *S.pun.* (A0A0L0HTG5); *Rozella allomycis*, *R.all.* (A0A075AZK2).

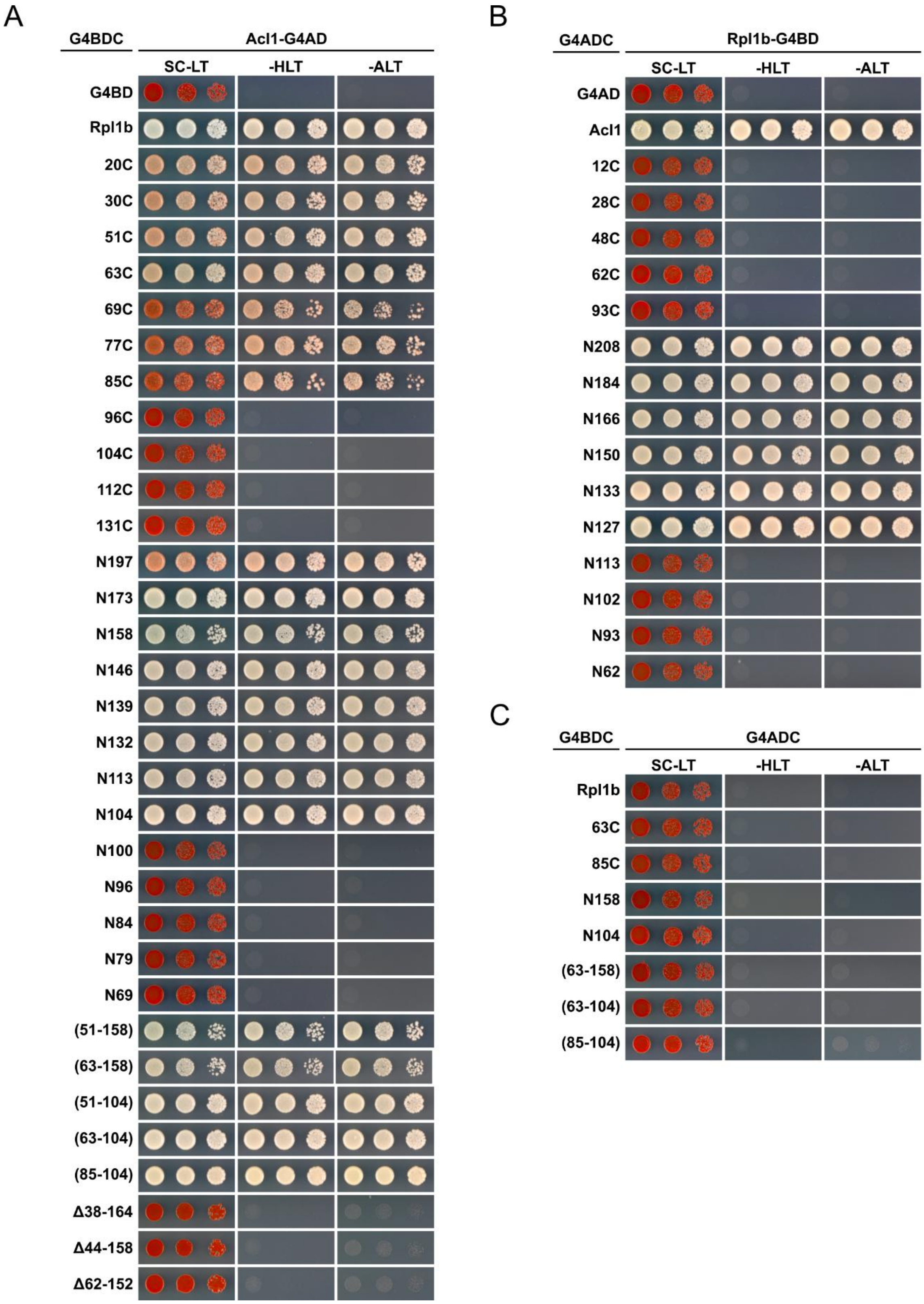

Supplementary Figure S12. Acl1 interacts via its ankyrin repeat domain with the second domain of Rpl1. (A, B) Complete data set for the mapping of the minimal respective interaction-mediating regions on Rpl1 **(A)** and Acl1 **(B)** by Y2H assays. **(C)** Negative control Y2Hs showing that the indicated, C-terminally G4BD-tagged Rpl1 variants do not self-activate the reporter genes when assayed together with the non-fused G4AD. Related to the data shown in Fig. 5A and B.

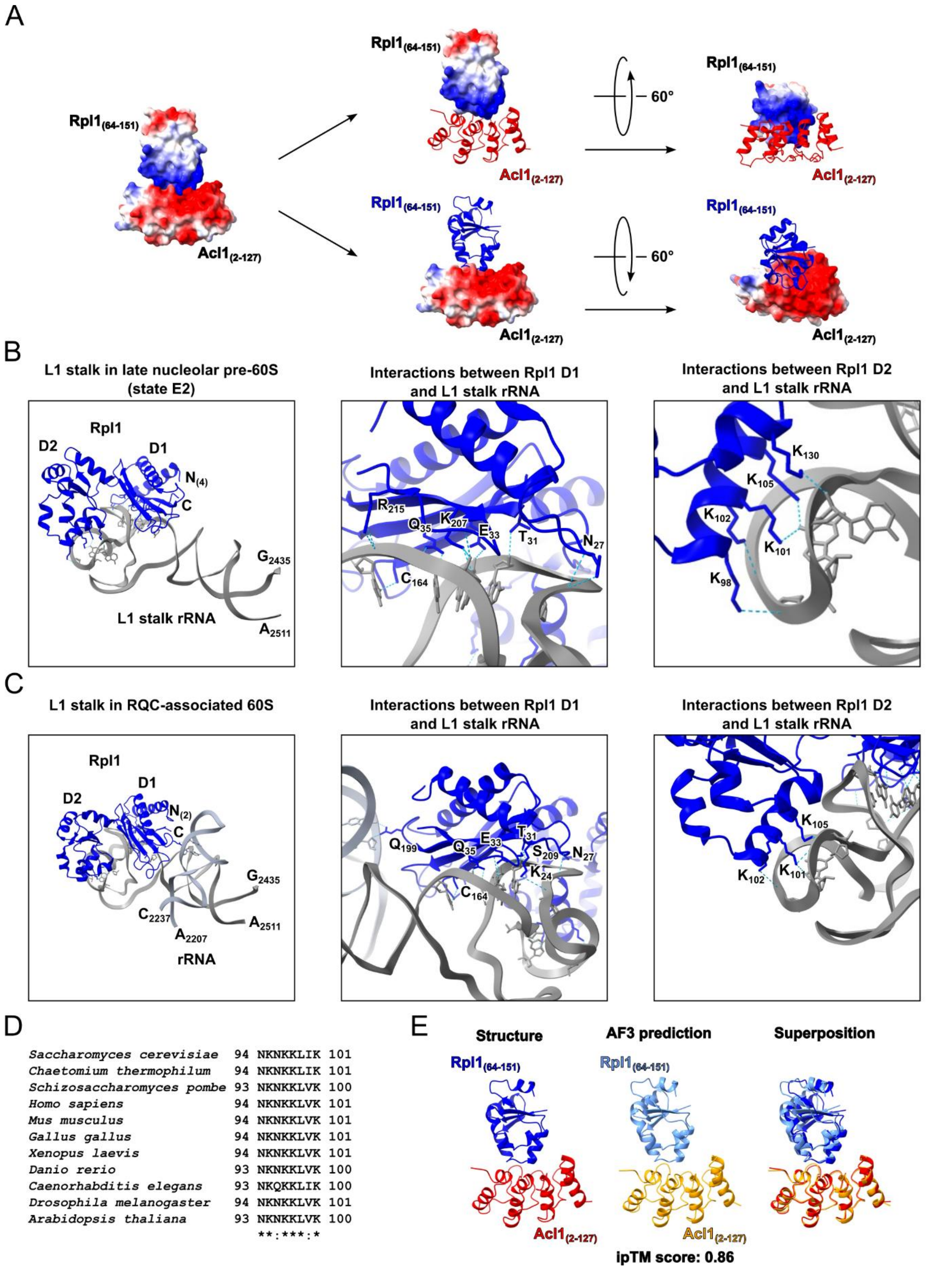

Supplementary Figure S13. (A) Representation of the electrostatic surface potential of the X-ray co-structure of the ankyrin repeat domain of Acl1 (residues 2-127) in complex with the second structural domain of Rpl1 (residues 64-151) with either Acl1 (in red) or Rpl1 (in blue) in cartoon representation to reveal the respective negatively (Acl1) or positively (Rpl1) charged interaction interfaces. **(B, C)** Representation of the interactions of Rpl1 with the L1 stalk rRNA as observed in the cryo-EM structure

of: **(B)** the late nucleolar state E2 pre-60S particle (PDB: 7R7C [8]) and **(C)** an RQC-associated 60S subunit (PDB: 8AGX [27]). **(D)** The Acl1-interacting residues of Rpl1 are conserved in Rpl1 orthologues in metazoans, plants, and other fungi, as shown here for Rpl1 of *Chaetomium thermophilum* (UniProt: G0S4Z9), *Schizosaccharomyces pombe* (O74836), *Homo sapiens* (P62906), *Mus musculus* (P53026), *Gallus gallus* (F6SU35), *Xenopus laevis* (Q7ZYS8), *Danio rerio* (Q6PC69), *Caenorhabditis elegans* (Q9N4I4), *Drosophila melanogaster* (Q9VTP4), and *Arabidopsis thaliana* (P59230). **(E)** Cartoon representation of the ankyrin repeat domain of Acl1 (residues 2-127) in complex with the second structural domain of Rpl1 (residues 64-151) as observed in our experimental X-ray co-structure or the AlphaFold3 model (ipTM score 0.86). The superposition of the two structures reveals their high similarity.

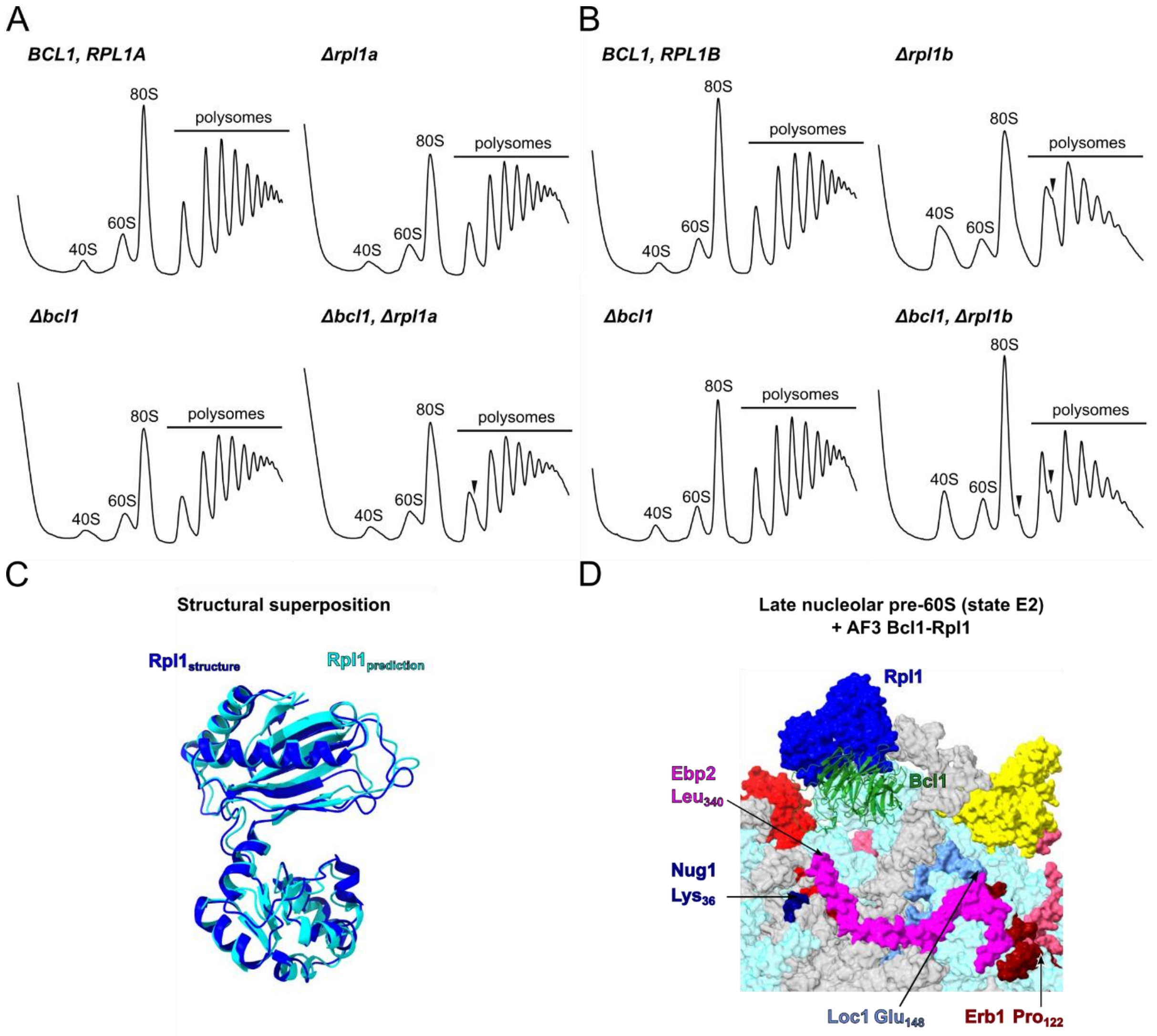

Supplementary Figure S14. (A, B) Polysome profiles of wild-type (*BCL1*, *RPL1A*), Δ*rpl1a*, Δ*bcl1*, and Δ*bcl1*/Δ*rpl1a* cells **(A)**, and of wild-type (*BCL1*, *RPL1B*), Δ*rpl1b*, Δ*bcl1*, and Δ*bcl1*/Δ*rpl1b* cells **(B)** grown at 30ºC in YPD medium. **(C)** Cartoon representation of the superposed predicted (extracted from the AlphaFold3 model of the Bcl1-Rpl1 complex) and observed (extracted from the late nucleolar state E2 pre-60S particle (PDB: 7NAC [8]) Rpl1 structures. **(D)** Close-up view of the superposition (shown in Fig. 8D) of the AlphaFold3 model of the Bcl1-Rpl1 complex onto Rpl1 within the late nucleolar state E2 pre-60S particle (PDB: 7NAC [8]), highlighting the first resolved residue of Erb1 and the last resolved residue of Ebp2, Nug1, and Loc1.

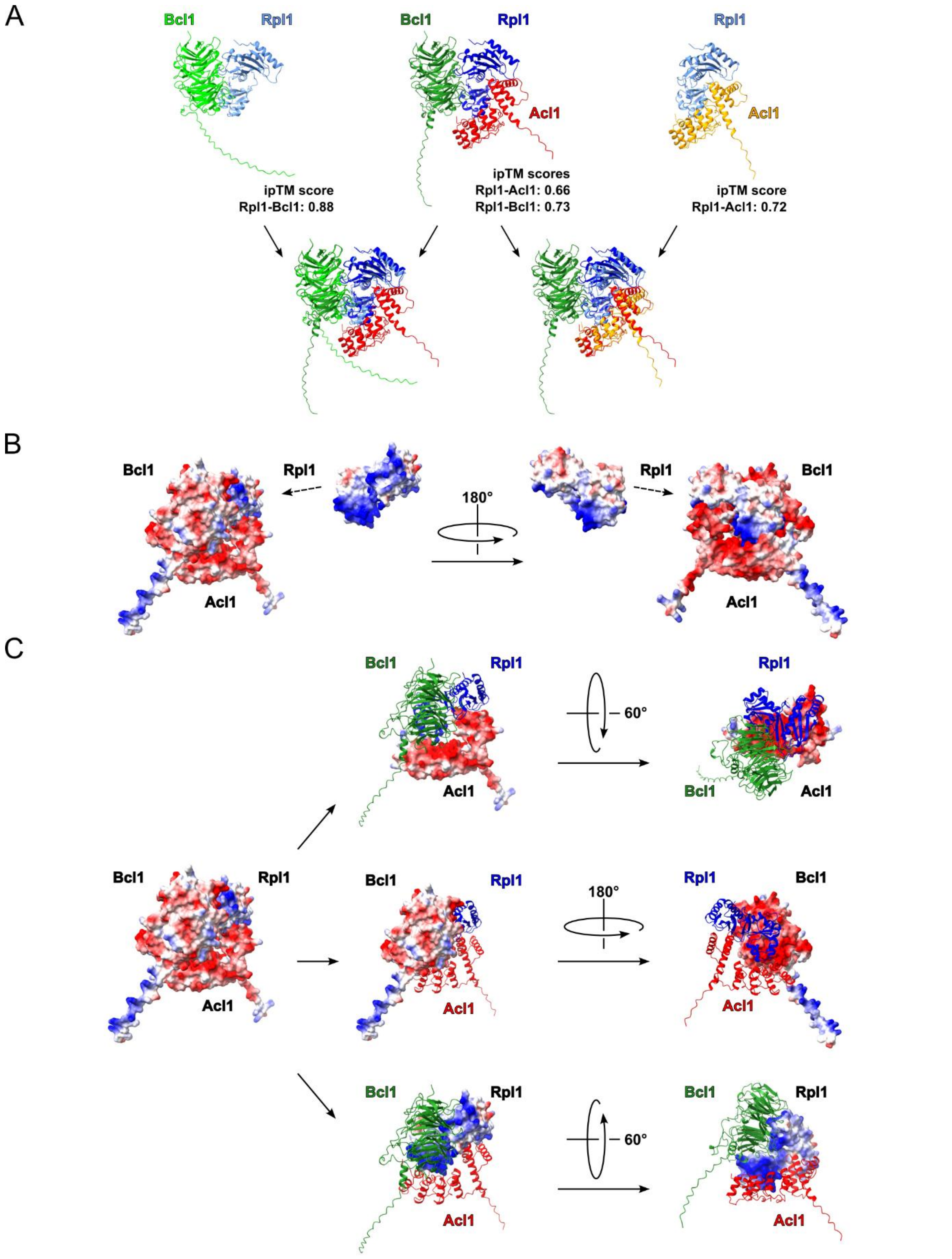

Supplementary Figure S15. (A) AlphaFold3 models of the Bcl1-Rpl1 (upper left), the Acl1-Rpl1-Bcl1 (upper middle), and the Acl1-Rpl1 (upper right) complexes. The lower panels show the superposition of the predicted Bcl1-Rpl1 or Acl1-Rpl1 complex with the predicted trimeric complex. The ipTM scores of the individual binary interactions are indicated. **(B, C)** Representation of the predicted electrostatic surface potential of the trimeric Acl1-Rpl1-Bcl1 complex (AlphaFold3 model)

(B) with either Bcl1 and Rpl1, Acl1 and Rpl1, or Acl1 and Bcl1 in cartoon representation to reveal the respective negatively (Acl1 and Bcl1) or positively (Rpl1) charged interaction interfaces **(C)**.

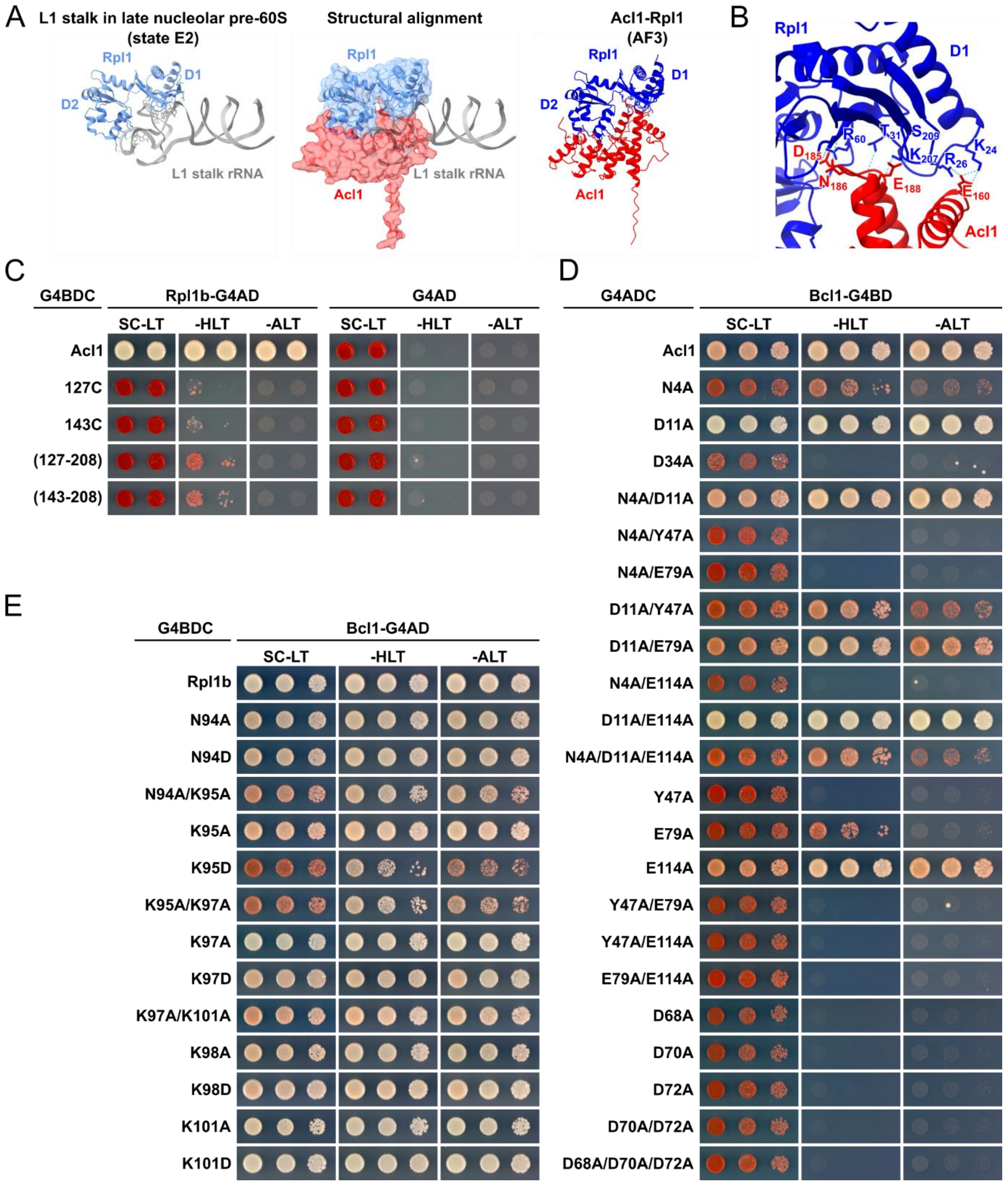

Supplementary Figure S16. (A) Interaction between Rpl1 (light blue) and the L1 stalk rRNA (grey) as observed on the late nucleolar state E2 pre-60S particle (left panel; PDB: 7R7C [8]). AlphaFold3 model of the Acl1-Rpl1 complex (right panel). Superposition of the AlphaFold3 model of the Acl1-Rpl1 complex and the Rpl1-bound L1 stalk rRNA (middle panel), revealing that Acl1 shields the rRNA-binding surfaces of Rpl1. **(B)** Close-up view of the predicted interaction between residues of the predicted three-helix fold within Acl1's C-terminal part and the first structural domain (D1) of Rpl1. **(C)** Y2H interaction assays between Rpl1 and full-length Acl1 or the indicated C-terminal segments of Acl1 (127C, residues 127-222; 143C, residues 143-222). The right panel shows the negative control Y2Hs with the non-fused G4AD, revealing that the indicated, C-terminally G4BD-tagged Acl1 variants do not self-activate the reporter genes. **(D, E)** Y2H interaction assays between Bcl1 and the indicated

Acl1 **(D)** or Rpl1 **(E)** mutant variants. Complete data set related to the data shown in Fig. 9F and G. Single-letter abbreviations for the amino acid residues are as follows: A, Ala; D, Asp; E, Glu; K, Lys; N, Asn; Y, Tyr.

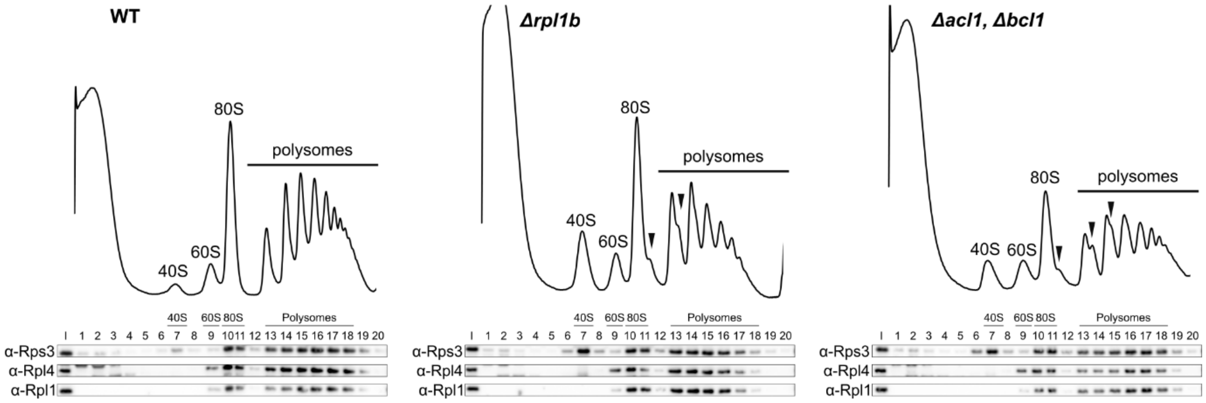

Supplementary Figure S17. Second experimental replicate of the sucrose gradient centrifugation and fractionation experiment shown in Fig. 10E that was used for the bar graph showing the average and standard deviation (n=2) of the normalized Rpl1/Rpl4 ratios in the 60S, 80S, and polysomal fractions of wild-type, Δ*rpl1b*, and Δ*acl1*/Δ*bcl1* cells in Fig. 10F.

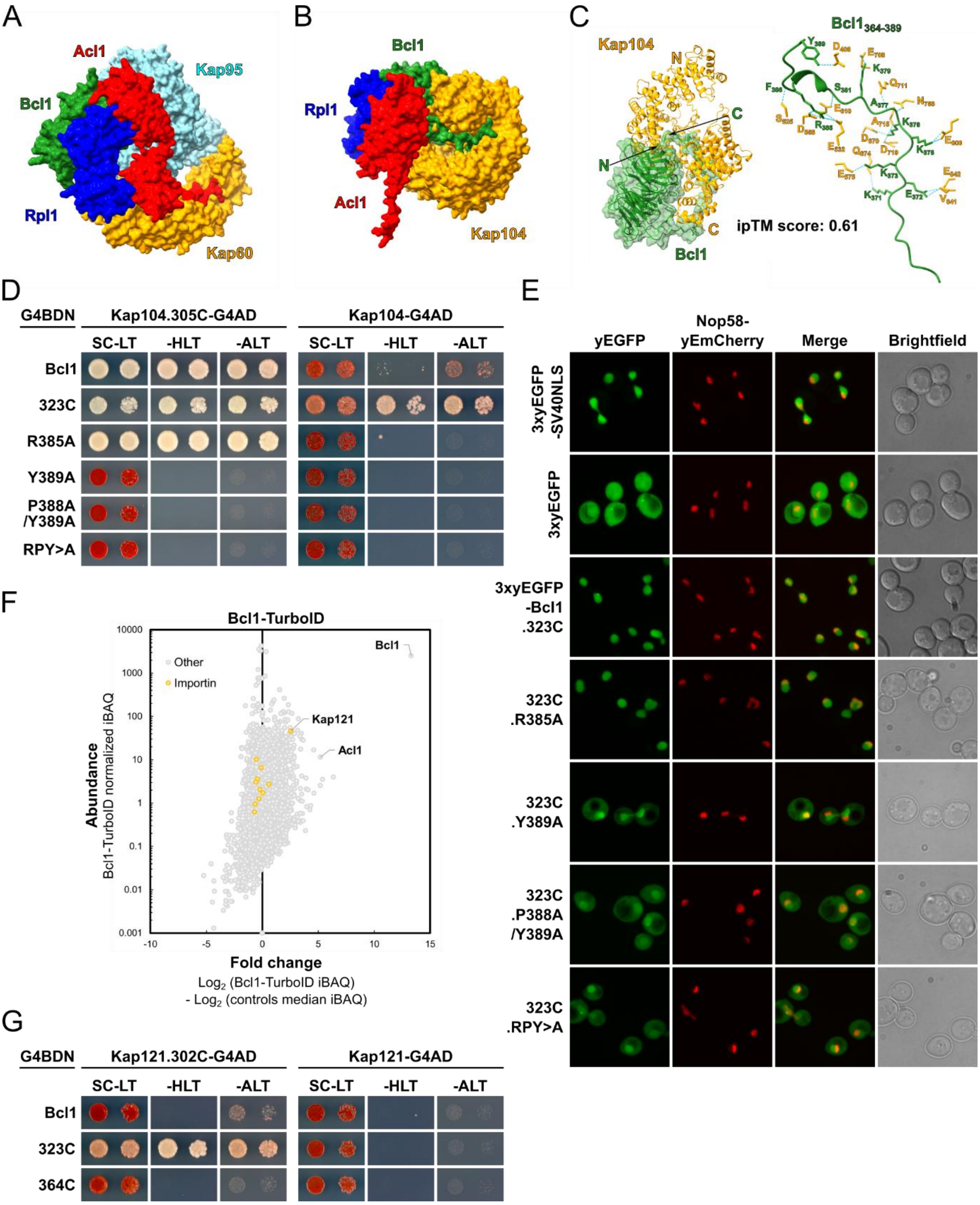

Supplementary Figure S18. **Acl1 and Bcl1 contain functional NLSs for the interaction with distinct importins. (A, B)** Surface representation of the AlphaFold3 model of the pentameric Kap95-Kap60-Acl1-Rpl1-Bcl1 complex **(A)** and the tetrameric Kap104-Bcl1-Rpl1-Acl1 complex **(B)**. **(C)** AlphaFold3 model of the binary Kap104-Bcl1 complex in cartoon representation with Bcl1 in semi-transparent surface representation (left). Close-up view showing the molecular details of the predicted interaction between Kap104 and the C-terminal bPY-NLS of Bcl1, with interacting residues being labelled and the observed H-bonds being depicted as dotted lines (right). **(D)** Y2H interaction assays between N-terminally G4BD-tagged (G4BDN) Bcl1, Bcl1.323C, and the indicated mutant variants of Bcl1.323C and C-terminally G4AD-tagged full-length Kap104 (right) or the N-terminally truncated

Kap104.305C variant (left). Single-letter abbreviations for the amino acid residues are as follows: A, Ala; P, Pro; R, Arg; Y, Tyr. **(E)** The nuclear targeting activity of Bcl1.323C and the indicated mutant variants thereof was assessed by fluorescence microscopy in cells grown at 30ºC in SC-Leu medium. The 3xyEGFP and 3xyEGFP-SV40NLS control proteins and the N-terminally 3xyEGFP-tagged Bcl1.323C fusion proteins were expressed from plasmid under the control of the *ADH1* promoter in cells expressing the nucleolar marker protein Nop58-yEmCherry from the genomic locus. **(F)** Graphical representation of the TurboID result obtained with C-terminally TurboID-tagged Bcl1 (Bcl1-TurboID). The Bcl1 bait protein, Acl1, and the enriched importin Kap121 are written in bold; orange dots highlight the location of importins on the graph. **(G)** Y2H interaction assays between N-terminally G4BD-tagged (G4BDN) Bcl1, Bcl1.323C, and Bcl1.364C and C-terminally G4AD-tagged full-length Kap121 (right) or the N-terminally truncated Kap121.302C variant (left).

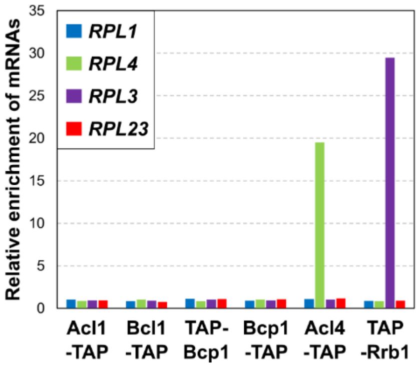

Supplementary Figure S19. Co-translational capturing assay. The DCs Acl1 (Acl1-TAP), Bcl1 (Bcl1-TAP), Bcp1 (TAP-Bcp1 and Bcp1-TAP), and, as positive controls, Acl4 (Acl4-TAP) and Rrb1 (TAP-Rrb1) were affinity purified (IgG-Sepharose pull-down) from extracts of cycloheximide-treated cells and the associated RNA was isolated from the TEV eluates. Each of the six DC purifications was assessed for their content of the four RPG mRNAs (*RPL1*, *RPL3*, *RPL4*, and *RPL23*) by real-time qRT-PCR. For each cDNA, real-time qPCRs were performed in triplicates. The bar graph shows the relative enrichment of the four RPG mRNAs in the six DC purifications.

Supplementary Table S1: Data collection and refinement statistics

	**Rpl1(63-158)-Acl1.N127**
**Data collection**	
X-ray source	ESRF, Grenoble<br>ID30A-3 (MASSIF-3)
Detector	Eiger1 X 4M
Wavelength (Å)	0.967697
Space group	$P6_522$
Cell dimensions (*a*, *b*, *c* (Å))	101.65, 101.65, 108.13
Resolution (Å)*	46.07-2.55 (2.70-2.55)
Total reflections	445071
Multiplicity	39.52
Unique reflections	11261
Completeness (%)	100.0 (100.0)
R_{meas} (%)	29.5 (222.0)
$CC_{1/2}$	99.8 (52.3)
$I/\sigma(I)$	17.87 (2.78)
Mosaicity (°)	0.221
Wilson *B*-factor (Å^2)	57.24
**Refinement**	
Resolution (Å)	46.07-2.55
R_{work}, R_{free}	0.195, 0.242
Reflections (working, test set)	10698, 563
Completeness for range (%)	100.0
r.m.s.d. from ideal	
Bond lengths (Å)	0.011
Bond angles (°)	1.190
Total number of atoms	1663
Mean *B* value (Å^2)	63.94

*Values in parentheses are for highest-resolution shell

Supplementary References

1. Kressler, D., Roser, D., Pertschy, B. *et al.* (2008) The AAA ATPase Rix7 powers progression of ribosome biogenesis by stripping Nsa1 from pre-60S particles. *J Cell Biol*. 181; 935-944. https://doi.org/10.1083/jcb.200801181.
2. Kater, L., Thoms, M., Barrio-Garcia, C. *et al.* (2017) Visualizing the Assembly Pathway of Nucleolar Pre-60S Ribosomes. *Cell*. 171; 1599-1610 e1514. https://doi.org/10.1016/j.cell.2017.11.039.
3. Sanghai, Z.A., Miller, L., Molloy, K.R. *et al.* (2018) Modular assembly of the nucleolar pre-60S ribosomal subunit. *Nature*. 556; 126-129. https://doi.org/10.1038/nature26156.
4. Ismail, S., Flemming, D., Thoms, M. *et al.* (2022) Emergence of the primordial pre-60S from the 90S pre-ribosome. *Cell Rep*. 39; 110640. https://doi.org/10.1016/j.celrep.2022.110640.
5. de la Cruz, J., Lacombe, T., Deloche, O. *et al.* (2004) The putative RNA helicase Dbp6p functionally interacts with Rpl3p, Nop8p and the novel trans-acting Factor Rsa3p during biogenesis of 60S ribosomal subunits in Saccharomyces cerevisiae. *Genetics*. 166; 1687-1699. https://doi.org/10.1534/genetics.166.4.1687.
6. Sailer, C., Jansen, J., Sekulski, K. *et al.* (2022) A comprehensive landscape of 60S ribosome biogenesis factors. *Cell Rep*. 38; 110353. https://doi.org/10.1016/j.celrep.2022.110353.
7. Cruz, V.E., Weirich, C.S., Peddada, N. *et al.* (2024) The DEAD-box ATPase Dbp10/DDX54 initiates peptidyl transferase center formation during 60S ribosome biogenesis. *Nat Commun*. 15; 3296. https://doi.org/10.1038/s41467-024-47616-7.
8. Cruz, V.E., Sekulski, K., Peddada, N. *et al.* (2022) Sequence-specific remodeling of a topologically complex RNP substrate by Spb4. *Nat Struct Mol Biol*. 29; 1228-1238. https://doi.org/10.1038/s41594-022-00874-9.
9. Mitterer, V., Thoms, M., Buschauer, R. *et al.* (2023) Concurrent remodelling of nucleolar 60S subunit precursors by the Rea1 ATPase and Spb4 RNA helicase. *Elife*. 12; e84877. https://doi.org/10.7554/eLife.84877.
10. Kater, L., Mitterer, V., Thoms, M. *et al.* (2020) Construction of the Central Protuberance and L1 Stalk during 60S Subunit Biogenesis. *Mol Cell*. 79; 615-628 e615. https://doi.org/10.1016/j.molcel.2020.06.032.
11. Ben-Shem, A., Garreau de Loubresse, N., Melnikov, S. *et al.* (2011) The structure of the eukaryotic ribosome at 3.0 A resolution. *Science*. 334; 1524-1529. https://doi.org/10.1126/science.1212642.
12. Sanghai, Z.A., Piwowarczyk, R., Vanden Broeck, A. *et al.* (2023) A co-transcriptional ribosome assembly checkpoint controls nascent large ribosomal subunit maturation. *Nat Struct Mol Biol*. 30; 594-599. https://doi.org/10.1038/s41594-023-00947-3.
13. Kargas, V., Castro-Hartmann, P., Escudero-Urquijo, N. *et al.* (2019) Mechanism of completion of peptidyltransferase centre assembly in eukaryotes. *Elife*. 8; e44904. https://doi.org/10.7554/eLife.44904.
14. Zhou, Y., Musalgaonkar, S., Johnson, A.W. *et al.* (2019) Tightly-orchestrated rearrangements govern catalytic center assembly of the ribosome. *Nat Commun*. 10; 958. https://doi.org/10.1038/s41467-019-08880-0.
15. Porras-Yakushi, T.R., Whitelegge, J.P., Miranda, T.B. *et al.* (2005) A novel SET domain methyltransferase modifies ribosomal protein Rpl23ab in yeast. *J Biol Chem*. 280; 34590-34598. https://doi.org/10.1074/jbc.M507672200.
16. Porras-Yakushi, T.R., Whitelegge, J.P. and Clarke, S. (2007) Yeast ribosomal/cytochrome c SET domain methyltransferase subfamily: identification of Rpl23ab methylation sites and recognition motifs. *J Biol Chem*. 282; 12368-12376. https://doi.org/10.1074/jbc.M611896200.
17. Yeh, M.C., Hsu, N.H., Chu, H.Y. *et al.* (2024) Dual protection by Bcp1 and Rkm1 ensures incorporation of uL14 into pre-60S ribosomal subunits. *J Cell Biol*. 223; 10171-10176. https://doi.org/10.1083/jcb.202306117.
18. Kosugi, S., Hasebe, M., Tomita, M. *et al.* (2009) Systematic identification of cell cycle-dependent yeast nucleocytoplasmic shuttling proteins by prediction of composite motifs. *Proc Natl Acad Sci U S A*. 106; 10171-10176. https://doi.org/10.1073/pnas.0900604106.

19. Abramson, J., Adler, J., Dunger, J. *et al.* (2024) Accurate structure prediction of biomolecular interactions with AlphaFold 3. *Nature*. 630; 493-500. https://doi.org/10.1038/s41586-024-07487-w.
20. Stewart, M. (2007) Molecular mechanism of the nuclear protein import cycle. *Nat Rev Mol Cell Biol*. 8; 195-208. https://doi.org/10.1038/nrm2114.
21. Homma, K., Terui, S., Minemura, M. *et al.* (1998) Phosphatidylinositol-4-phosphate 5-kinase localized on the plasma membrane is essential for yeast cell morphogenesis. *J Biol Chem*. 273; 15779-15786. https://doi.org/10.1074/jbc.273.25.15779.
22. Audhya, A. and Emr, S.D. (2003) Regulation of PI4,5P2 synthesis by nuclear-cytoplasmic shuttling of the Mss4 lipid kinase. *EMBO J*. 22; 4223-4236. https://doi.org/10.1093/emboj/cdg397.
23. Sekulski, K., Cruz, V.E., Weirich, C.S. *et al.* (2023) rRNA methylation by Spb1 regulates the GTPase activity of Nog2 during 60S ribosomal subunit assembly. *Nat Commun*. 14; 1207. https://doi.org/10.1038/s41467-023-36867-5.
24. Li, Z., Chen, S., Zhao, L. *et al.* (2023) Nuclear export of pre-60S particles through the nuclear pore complex. *Nature*. 618; 411-418. https://doi.org/10.1038/s41586-023-06128-y.
25. Ben-Shem, A., Jenner, L., Yusupova, G. *et al.* (2010) Crystal structure of the eukaryotic ribosome. *Science*. 330; 1203-1209. https://doi.org/10.1126/science.1194294.
26. UniProt, C. (2023) UniProt: the Universal Protein Knowledgebase in 2023. *Nucleic Acids Res*. 51; D523-D531. https://doi.org/10.1093/nar/gkac1052.
27. Tesina, P., Ebine, S., Buschauer, R. *et al.* (2023) Molecular basis of eIF5A-dependent CAT tailing in eukaryotic ribosome-associated quality control. *Mol Cell*. 83; 607-621 e604. https://doi.org/10.1016/j.molcel.2023.01.020.
